# Improved Synthesis of Effective 3-(Indolin-6-yl)-4-(*N*-pyrazole-sulfonamide)-1*H*-pyrrolo[2,3-*b*]pyridine-Based Inhibitors of NADPH Oxidase 2

**DOI:** 10.3390/ijms26083647

**Published:** 2025-04-12

**Authors:** Konstantin V. Potapov, Dmitry N. Platonov, Alexander Yu. Belyy, Maxim A. Novikov, Yury V. Tomilov, Anastasia A. Anashkina, Kristina A. Mukhina, Olga I. Kechko, Pavel N. Solyev, Roman A. Novikov, Alexander A. Makarov, Vladimir A. Mitkevich

**Affiliations:** 1 Engelhardt Institute of Molecular Biology of the Russian Academy of Sciences, 119991 Moscow, Russia; kospotapov@yandex.ru (K.V.P.); manovikov@ioc.ac.ru (M.A.N.); anastasia.a.anashkina@mail.ru (A.A.A.); kristina.mukhina@gmail.com (K.A.M.); olga.kechko@gmail.com (O.I.K.); aamakarov@eimb.ru (A.A.M.); mitkevich@gmail.com (V.A.M.); 2 Zelinsky Institute of Organic Chemistry of the Russian Academy of Sciences, 119991 Moscow, Russia; dmplatonov@rambler.ru (D.N.P.); duospirit@gmail.com (A.Y.B.); tom@ioc.ac.ru (Y.V.T.)

**Keywords:** reactive oxygen species, NOX2 inhibitor, Alzheimer’s disease, GSK2795039, microglial cells

## Abstract

NADPH oxidase enzymes (NOXs) are a family of enzymes generating superoxide, which form reactive oxygen species. NOX2 activity is a causative agent for the progression of many diseases: neurodegenerative, cardiovascular, immune dysregulations, and even hereditary diseases and cancer. Administering antioxidants helps in inhibiting NOX2 activity; however, the development of selective inhibitors may provide greater improvement in the therapy of diseases. Here, an optimized synthesis of two most promising NOX2 inhibitors based on the 3-(indolin-6-yl)-4-(*N*-pyrazole-sulfonamide)-1*H*-pyrrolo [2,3-*b*]pyridine structure, namely, GSK2795039 and NCATS-SM7270, and an isomeric derivative of the same class, IMBIOC-1, is reported. The new modified procedures simplify the isolation, reduce byproduct formation, and improve the yields in 0.1–1 g scale preparations. Molecular modeling of the structures of NOX2 complexes with inhibitors validated their binding at the same site as NADPH, with IMBIOC-1 forming the largest number of intermolecular interactions with the NOX2 active site. Testing the effects of the compounds on amyloid beta-induced oxidative stress and toxicity in HMC3 microglial cells showed that all three inhibitors completely prevented the pathological amyloid-beta effect. At the same time, NCATS-SM7270 and IMBIOC-1 provided a stronger protective effect on microglial cell survival than GSK2795039, which allowed us to assert the potential of those compounds as neuroprotective agents.

## 1. Introduction

Neuroinflammation and oxidative stress are major pathological processes associated with many neurodegenerative diseases such as Alzheimer’s disease (AD), Parkinson’s disease (PD), status epilepticus, and amyotrophic lateral sclerosis [1]. Elevated levels of reactive oxygen species (ROS) cause brain tissue damage and trigger a secondary cascade of ROS production, which increases neuroinflammation and neurodegeneration. Suggested antioxidant therapy based on scavenging and removing free radicals disrupts cell signaling and remains unsuccessful in clinical trials.

The inhibition of ROS generation rather than their complete elimination is an alternative way to suppress oxidative stress [2]. A major source of ROS in the brain is NADPH oxidase enzymes—transmembrane proteins that transport electrons across biological membranes to reduce oxygen to superoxide. Among them, the greatest contribution to the development of neuropathology is attributed to microglial NADPH oxidase 2 (NOX2) [3,4]. Microglia activation leads to NOX2 upregulation, which significantly contributes to the development of the two most prevalent neurodegenerative diseases, AD and PD [5]. NOX2 has been shown to be a key regulator of microglia-mediated neurotoxicity [5]. NOX2 knockout blocks the development of oxidative stress and behavioral deficits in AD [6] and also has a neuroprotective and anti-inflammatory effect in PD [7].

**Figure 1:**
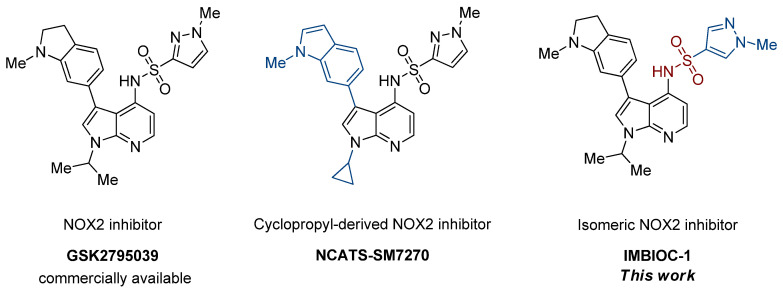
Examples of the inhibitors of NADPH oxidase 2 (NOX2) based on 3-(indolin-6-yl)-4-(*N*-pyrazole-sulfonamide)-1*H*-pyrrolo[2,3-*b*]pyridine skeleton: GSK2795039 [12,18], NCATS-SM7270 [3], and a newly found inhibitor isomeric to GSK2795039.

Given the importance of the target, the number of different candidates for inhibitors of NOX2 is growing [1,8,9,10,11,12]. One of the most promising structural types of NOX2 inhibitors is GSK2795039, based on the 3-(indolin-6-yl)-4-(*N*-pyrazole-sulfonamide)-1*H*-pyrrolo[2,3-*b*]pyridine skeleton (Figure 1), which possesses good properties for in vivo studies [12,13,14,15]. GSK2795039 is known to cross the blood–brain barrier [12] and also successfully prevents amyloid-beta (Aβ)-driven AD pathogenesis [16]. This structural type has been used as a basis for a number of other derivatives [17], which allowed an even more effective cyclopropyl-substituted derivative (NCATS-SM7270) to be discovered (Figure 1). However, only one parent compound GSK2795039 is currently commercially available for research purposes [3]. Since these pharmacophores have been patented, when it comes to larger than several mg scale productions, their synthesis description actually lacks the majority of experimental data, or it is fragmented and incomplete to reproduce even in the articles relevant to the topic. For example, the key steps (including final stages of palladium cross-coupling, requiring fairly harsh conditions) give poor yields [17].

Thus, the goal of our study was to improve the synthesis methodology of NOX2 inhibitors of the GSK2795039 type for better yields, more convenient conditions, and available reagents, as well as to validate the compound effect on microglia cells. The synthesis was optimized to adjust reaction conditions to be tolerated by the oxidation-sensitive intermediate products and to prevent several purification cycles using preparative HPLC chromatography for the target product isolation. The efficacy of the synthesized inhibitors in preventing the pathological effects of Aβ was validated on human microglial cells.

## 2. Results and Discussion

### 2.1. Chemical Synthesis

To start our investigation, we revisited the synthetic route of GSK2795039. In general, our synthetic scheme follows the original route to obtain the product, since the straightforward assembly of the three heterocyclic moieties by two sequential cross-couplings is well justified. Nevertheless, some conditions and functional groups used in original stages needed to be re-screened and were significantly changed, especially those used at cross-coupling stages. We performed a number of stages with gram and sub-gram amounts of reagents, and the final stages were adjusted to be scalable and reproducible, which was tested in several experiments. For specific examples of compounds, we focused on the two most active inhibitors GSK2795039 and NCATS-SM7270 (Figure 1). The redesigned synthesis allowed us to broaden the series and obtain an undescribed analog of the isomeric sulfopyrazoline product IMBIOC-1. This candidate turned out to be even more functionally active, more cost-effective than GSK2795039, and a little more accessible from a synthetic point of view.

**Figure 2:**
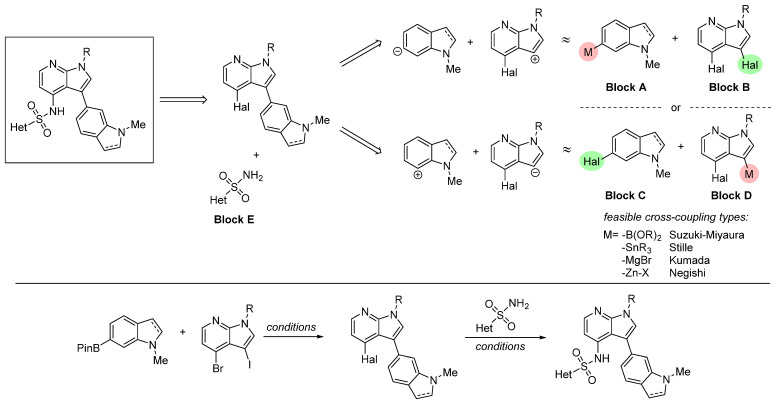
General retrosynthetic analysis for synthesis of the GSK2795039-type NOX2 inhibitors (top) and the proposed pathway of synthesis (below).

Retrosynthetic analysis of the GSK2795039-type inhibitors revealed the most optimal pathway for synthesis as the step-by-step assembly from three synthons involved in two consecutive cross-coupling reactions: the Buchwald–Hartwig reaction with the formation of the C(aryl)–N bond between azaindole and sulfonamide fragments and the C(aryl)–C(aryl) bond cross-coupling, which allows the azaindole to be linked to indole or the indoline core (Figure 2).

The first cross-coupling can be achieved via two reverse-polarized transformations (Figure 2). In one case, the azaindole structural block **D** can be used as the metal–organic component, and the indole or indoline block **C** acts as the halide. This option has a number of drawbacks; the main one is the complexity of the synthesis of structure **D** due to the presence of both a halogen atom and a metal in one molecule. Another option, where the polarity is reversed and an indole or indoline block **D** acts as the metal–organic reagent, is much more convenient for the synthesis. Due to high nucleophilicity and sensitivity of the compounds to moisture and air, it was decided to abandon the use of organomagnesium and organozinc components in the M-substituent. Synthesis and purification of the products were predicted to be more achievable with the organoboron-substituted block **A**, which can be obtained from the commercially available 6-brominol in a common two-step sequence (alkylation and subsequent borylation) (Figure 2, bottom). Then, at the second cross-coupling reaction between the obtained halide and the sulfonamide block **E**, the formation of the target NOX inhibitor is expected. Thus, the developed method of the synthesis of GSK2795039-type inhibitors involves two consecutive processes of cross-coupling with block **C** containing two halogen atoms. Block **B** was designed to contain bromide and iodide substituents, which allows us to increase the regioselectivity of reactions varying the conditions of cross-coupling reactions and therefore potentially avoid the side process occurrence.

**Scheme 1:**
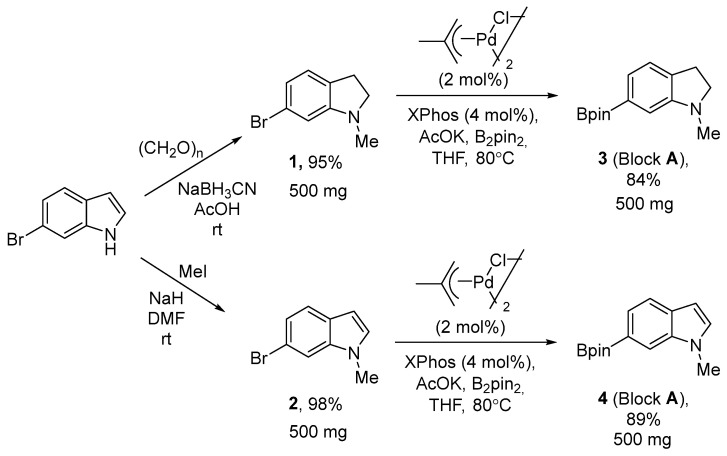
Synthesis of (Bpin)-indoline and (Bpin)-indole derivatives (blocks **A**).

The total synthesis of NOX inhibitors started with the preparation of building blocks **A**, **B** and **E**. Indoline **1** was prepared according to the described procedure [18] by simultaneous reduction with NaBH_3_CN and monomethylation of the nitrogen atom with paraformaldehyde in glacial acetic acid solution in 95% yield. Indole **2** was obtained by the direct methylation of 6-bromoindol with methyl iodide using NaH as a base in 98% yield [19]. The key step in the synthesis of blocks **A** is the Miyaura borylation of bromindoline **1** or bromindol **2** with B_2_pin_2_ in the presence of the allyl palladium complex with XPhos as a ligand. The reactions were carried out under standard conditions, heating in THF in the presence of potassium acetate as a base. The yields of target boronic esters **3** and **4** exceeded 80% (Scheme 1).

**Scheme 2:**
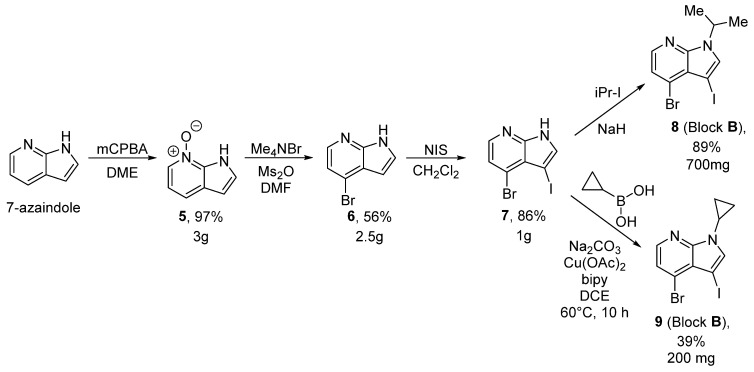
Synthesis of dihalogenoindole-based building blocks **C**.

Block **B** was prepared according to the described procedure [17] from commercially available 7-azaindole in a four-step synthesis. In the first step, 7-azaindole was oxidized with *m*CPBA to the *N*-oxide **5**, which was purified by column chromatography and then converted into the bromo derivative **6**. Treatment of a solution of bromide **6** in CH_2_Cl_2_ with *N*-iodosuccinimide resulted in the formation of 4-bromo-3-iodoazaindole **7**. Block **B** (**8**) was obtained from derivative **7** by alkylation of the nitrogen atom of the pyrrole ring with isopropyl iodide using sodium hydride as a base. Block **B** (**9**), which cannot be prepared by the direct alkylation with cyclopropyl iodide or cyclopropyl bromide, was prepared by the Buchwald–Hartwig amination-type cross-coupling of azaindole **7** with cyclopropyl boronic acid in 39% yield (Scheme 2).

**Scheme 3:**
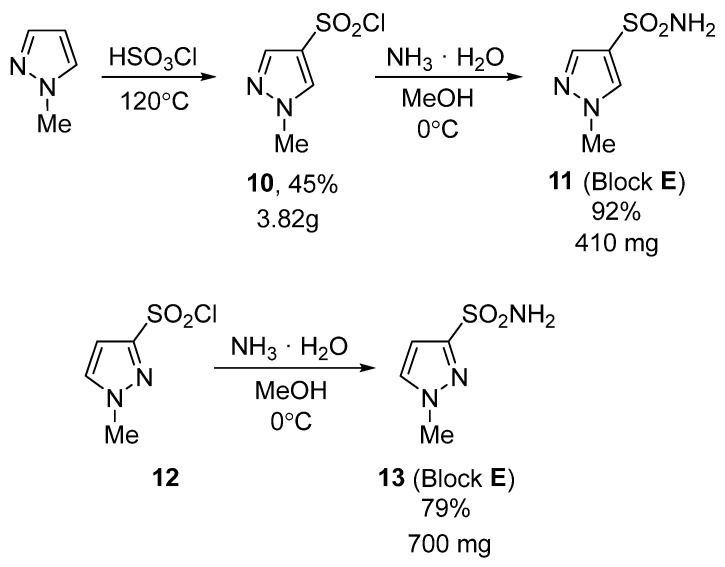
Synthesis of pyrazole-based building blocks **E**.

Sulfonamides **11** and **13** (blocks **E**) were synthesized according to the described methods from the corresponding sulfonyl chlorides **10** and **12** as a result of the reaction with aqueous ammonia in the presence of methanol, yielding 92% and 79%, respectively (Scheme 3) [20].

**Scheme 4:**
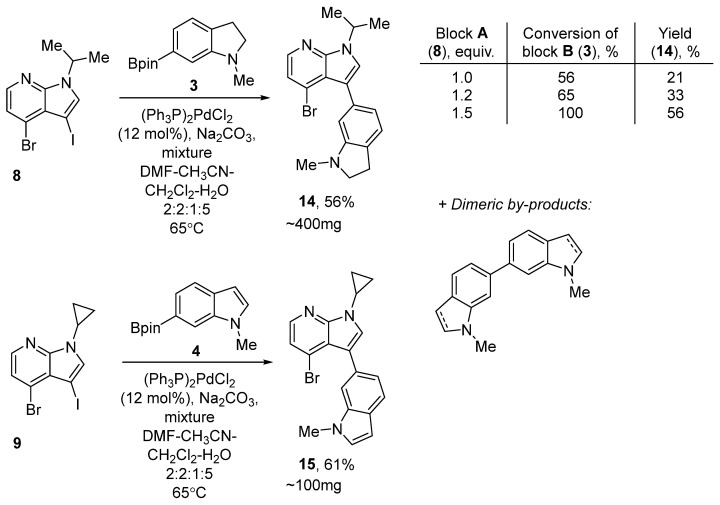
The Suzuki cross-coupling between blocks **A** and **B**.

The Suzuki coupling between block **A** and block **B** was carried out according to the classical procedure under (Ph_3_P)_2_PdCl_2_ catalysis in the presence of Na_2_CO_3_ as a base, as described in the original patent [17]. The process is stereospecific, and the products are formed in the form of single isomers **14** or **15** (Scheme 4). However, it was found that, under these conditions, a byproduct-forming dimerization of the boronate ester occurs with the formation of bisindole or bisindoline, while a significant amount of the unreacted azaindole remains in the reaction mass. The yield of the products in this case did not exceed 35%. During the optimization of the Suzuki cross-coupling conditions using **3** and **8** as model substrates, it was found that the complete conversion of block **B** is observed at least with a 1.5-fold excess of block **A** (Table 1). The yield of the target product **14** was increased more than twofold compared to the method presented in the original patent. Using these optimized conditions, a similar Suzuki coupling was further carried out for the cyclopropyl analog **9**, resulting in the product **15** formation with the isolated yield of 61%.

**Scheme 5:**
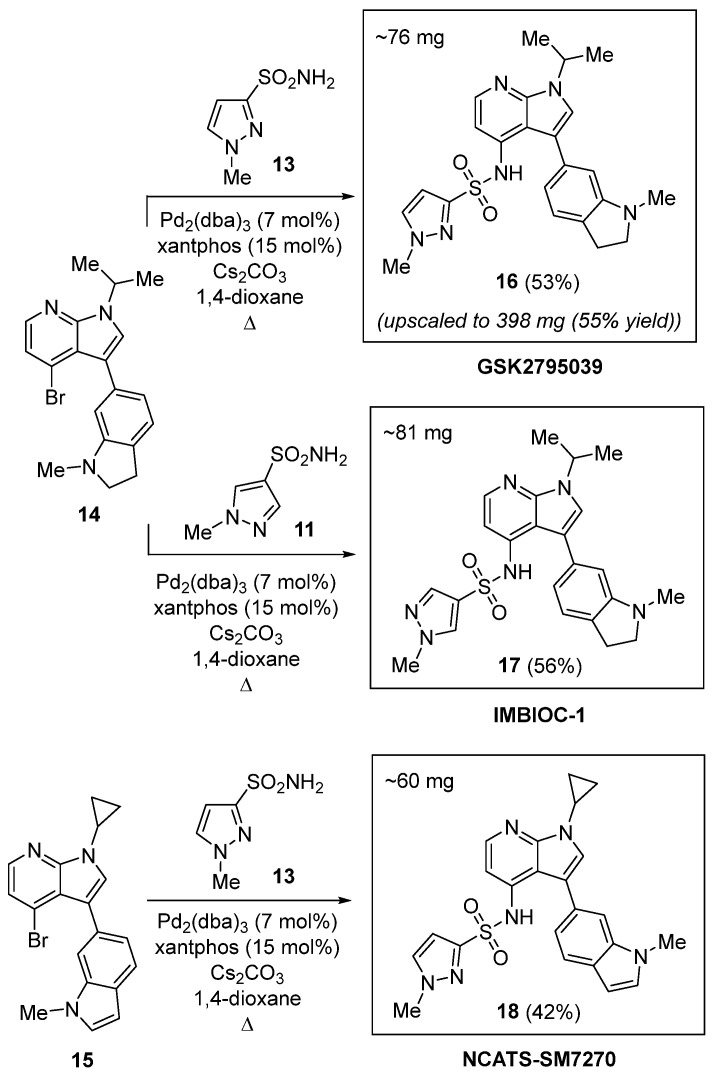
Final Buchwald–Hartwig cross-coupling for synthesis of all three NOX2 inhibitors.

Final cross-coupling to obtain the target compounds required the Buchwald–Hartwig reaction of **14** or **15** with the block **E** sulfonamides **11** or **13**. The procedure from the original patent [17] described that reaction to be carried out under microwave irradiation at 150 °C using Pd(OAc)_2_/xantphos as a catalyst system. However, these conditions caused significant complications. It was found that, under those harsh conditions, GSK2795039 was formed in a complex mixture, with the isolation of the target product being a challenging and laborious task, requiring several purification cycles via preparative HPLC. Other homologues—IMBIOC-1 and NCATS-SM7270—are not formed at all under these conditions. We tested several options and found out that replacing the catalyst with the Pd_2_(dba)_3_/xantphos system and setting the process without microwave irradiation under milder conditions at the boiling point of 1,4-dioxane provides much smoother reactions with a better scope and outcome. As a result, conventional column chromatography can be used to isolate a high-purity product in one passage (Scheme 5). Under the new conditions, GSK2795039 and IMBIOC-1 can be isolated with yields of 53% and 56%; similar universal conditions were used with the cyclopropyl analog to form the target NCATS-SM7270 in 42% yield.

To sum, we revisited the synthetic route to an effective NOX2 inhibitor based on 3-(indolin-6-yl)-4-(*N*-pyrazolesulfonamide)-1*H*-pyrrolo[2,3-*b*]pyridine structure—GSK2795039—optimized the procedures for its synthesis in 0.1–1 g scale quantities, reduced byproduct formation, increasing the product yield by almost two times compared to the original patent. We applied the optimized conditions for the synthesis of the second NOX2 inhibitor—NCATS-SM7270—and obtained a new derivative of the same class as a potential NOX2 inhibitor—IMBIOC-1.

### 2.2. Modeling of NOX2 Complexes with Inhibitors

The molecular mechanism of GSK2795039 and its analogs suggests that they bind to NOX2 at the NADPH interaction site and thus prevent its complex formation with the protein. However, to our knowledge, there are no structural data to support such a mechanism. Therefore, we used molecular modeling to generate structures of complexes of the synthesized inhibitors with NOX2 based on the recently obtained structure of the NOX2: NADPH complex [21].

**Figure 3:**
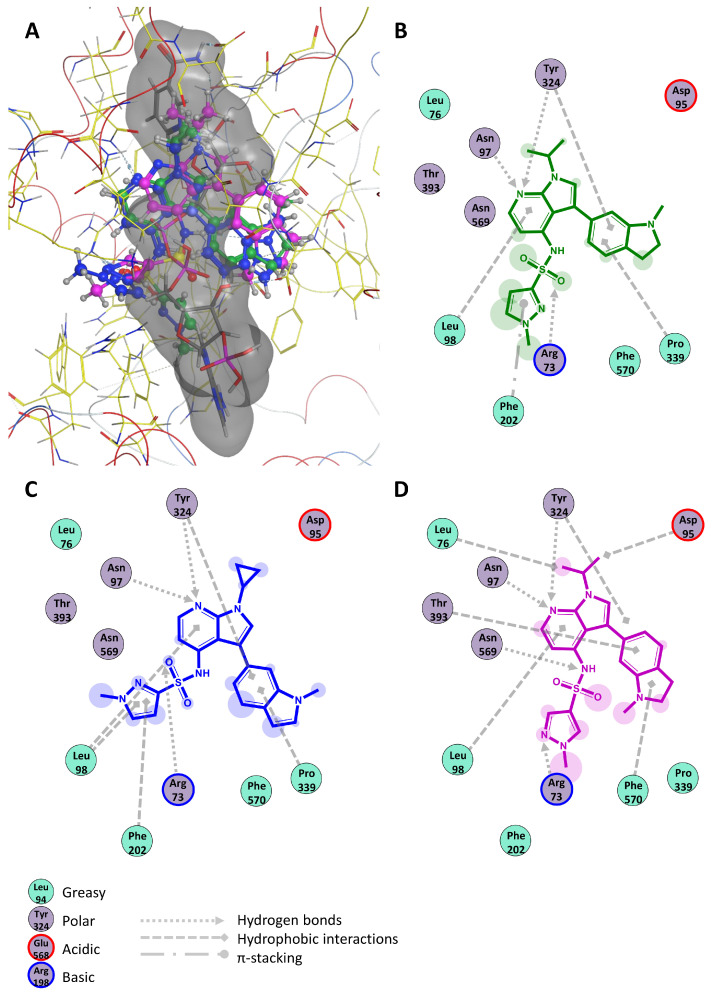
Binding sites of the inhibitors of GSK2795039, NCATS-SM7270, IMBIOC-1, and NADPH with NOX2: (**A**) The docking site of NADPH (gray area) and inhibitors GSK2795039 (green), NCATS-SM7270 (blue), and IMBIOC-1 (magenta). (**B**) Amino acid residues of the NOX2 interacting with GSK2795039 inhibitor. (**C**) Amino acid residues of NOX2 interacting with the NCATS-SM7270 inhibitor. (**D**) Amino acid residues of NOX2 interacting with the IMBIOC-1 inhibitor.

The NADPH binding site in NOX2 consists of the following amino acid residues: 203Glu, 341Thr, 354His, 358Val—forming hydrogen bonds, 570Phe—forming π-stacking interactions, and magnesium ion Mg^2+^ coordinated by 338His—forming salt bridges. Both blind and targeted methods of docking inhibitors to the NOX2 structure gave identical results, positioning the inhibitors in the NADPH binding site (Figure 3A).

The positions of all inhibitors (GSK2795039, NCATS-SM7270, IMBIOC-1) and NADPH are similar, indicating that inhibitor binding blocks the NADPH binding site. The inhibitor molecules arrange in a similar manner in the NOX2 binding site. The azaindole groups of GSK2795039 and NCATS-SM7270 are positioned exactly the same in the NOX2 binding site, while, in IMBIOC-1, it is shifted closer to 324Tyr and 393Thr residues (Figure 3B–D). Due to this, the indole group in IMBIOC-1 can form hydrophobic interactions with 324Tyr residue, rather than with 339Pro as in GSK2795039 and NCATS-SM7270. There is also a difference in the position of the pyrazole ring in IMBIOC-1, which forms a hydrogen bond with the side chain of 73Arg residue via the nitrogen atom. GSK2795039 and NCATS-SM7270 also form a hydrogen bond with 73Arg, but on the ligand side, with the oxygen atom of the sulfonyl group involved. In GSK2795039 and NCATS-SM7270, the pyrazole group forms hydrophobic interactions with residues 98Leu and 202Phe (π-stacking in the case of GSK2795039). The positions of the indole and azaindole fragments of GSK2795039 and NCATS-SM7270 in the NOX2 binding site are almost identical, and their pyrazole fragments are located perpendicular to each other. The bonds formed by the interaction of NOX2 amino acid residues and inhibitors are summarized in Table 1. IMBIOC-1 forms the largest number of hydrogen bonds and hydrophobic interactions with NOX2. Calculation of the binding energy shows that IMBIOC-1 has the highest affinity to NOX2, which, when forming a complex with NOX2, results in the Gibbs free energy change value of −8.85 kcal/mol. The obtained modeling results are in good agreement with the studies on the cellular model described below.

### 2.3. Effect on ROS Production and Cytotoxic Properties of NOX2 Inhibitors

Microglia are the main resident immune cells in the brain and play a critical role in the development of neuropathologies. In particular, pathogenic Aβ aggregates are recognized by microglial receptors, activate the cells, and trigger the immune response in AD [22]. Microglial activation is accompanied by increased oxidative stress [23], which, in turn, accelerates the development of pathology and increases its severity [24].

Recently, it was shown that GSK2795039 treatment reduced negative changes in mice brain and behavior caused by Aβ-driven pathology [17]. This evidence suggests a promising role of NOX2 inhibitors in treating Aβ-induced neuroinflammation and oxidative stress.

**Figure 4:**
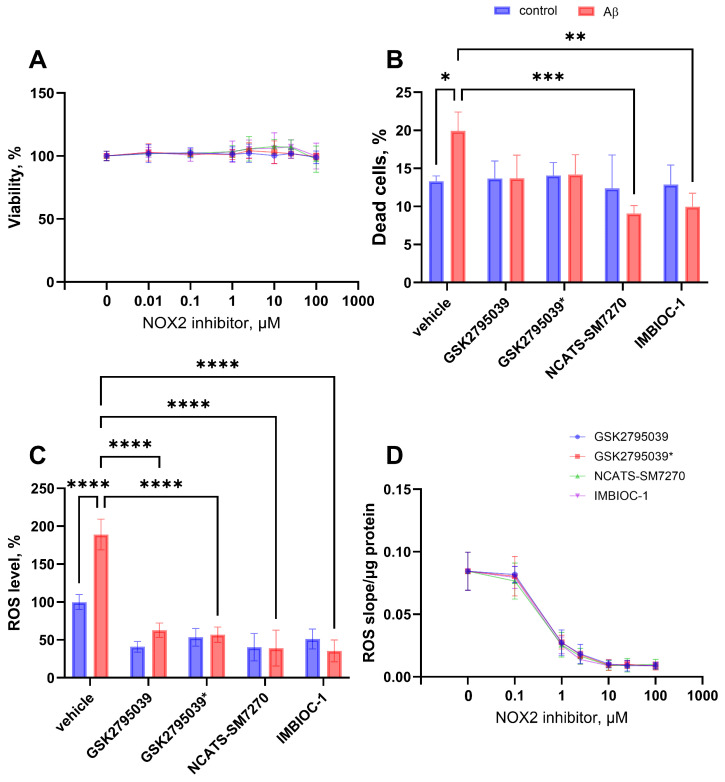
Effect of GSK2795039 (commercial), GSK2795039* (synthesized), NCATS-SM7270, and IMBIOC-1 on cell death and ROS level in HMC3 microglial cells in the absence and presence of Aβ: (**A**) MTT analysis of viability of HMC3 cells treated with NOX2 inhibitors for 24 h. (**B**) Flow cytometry analysis of dead cells in HMC3 population after 24 h incubation with 10 µM of Aβ and 25 µM inhibitors. (**C**) Flow cytometry analysis of ROS level in HMC3 cells treated with 10 µM of Aβ and 25 µM inhibitors for 24 h. (**D**) Determination of IC50 values for NOX2 inhibitors in HMC3 cells treated with 10 µM of Aβ for 24 h. Data are presented as mean ± SD of at least three independent experiments with triplicate measurements. * *p* < 0.05; ** *p* < 0.01; *** *p* < 0.001; **** *p* < 0.0001.

We compared the effect of four NOX2 inhibitors on cell viability and Aβ-induced oxidative stress in HMC3 human microglial cells. Toxicity of the inhibitors on microglial cells was investigated by a standard MTT assay, and no significant cytotoxicity was observed up to 100 μM of the compounds (Figure 4A). All inhibitors completely prevented HMC3 death caused by Aβ (Figure 4B). NCATS-SM7270 and IMBIOC-1 had a more pronounced protective effect on cell survival than GSK2795039.

Aβ induced oxidative stress in HMC3 by an 83% rise in ROS level (Figure 4C). All inhibitors effectively reduced Aβ-driven ROS production in the cells (Figure 4C). Half-maximal inhibitory concentration 50 (IC50) values for NOX2 inhibitors were around 0.5 μM and were equal for commercially available GSK2795039 (0.51 ± 0.15 μM) and synthesized GSK2795039 (0.50 ± 0.15 μM), NCATS-SM7270 (0.42 ± 0.14 μM), and IMBIOC-1 (0.46 ± 0.12 μM) (Figure 4D).

As expected, Aβ induced oxidative stress and toxicity in HMC3 cells. ROS level and the number of dead cells increased by 83% and 6%, respectively, in the presence of Aβ (Figure 4A,B). All inhibitors completely prevented the pathological Aβ effect on HMC3 cells. The effect of our synthesized GSK2795039 did not differ from the commercially available compound. At the same time, NCATS-SM7270 and IMBIOC-1 showed a stronger protective effect on the survival of microglial cells than GSK2795039 (Figure 4A).

## 3. Materials and Methods

### 3.1. Human Microglia Cell (HMC3) Culture

The human microglial clone 3 (HMC3) cell line was grown in minimum essential medium (MEM, Gibco, ThermoFisher Scientific, Waltham, MA, USA) containing 10% fetal bovine serum (Gibco, ThermoFisher Scientific, Waltham, MA, USA), 100 U/mL of penicillin and 100 µg/mL of streptomycin (Gibco, ThermoFisher Scientific, Waltham, MA, USA), sodium pyruvate and GlutaMax (Gibco, ThermoFisher Scientific, Waltham, MA, USA). Cells were grown in a humidified atmosphere of 5% CO_2_ at 37 °C.

### 3.2. Treatment with Aβ and NOX2 Inhibitors

Commercial GSK2795039 (GSKs, MedChemExpress, Monmouth Junction, NJ, USA) and chemically synthesized by us GSK2795039, NCATS-SM7270 or IMBIOC-1 were dissolved in sterile DMSO (Sigma-Aldrich, St. Louis, MO, USA) at a concentration of 10 mM. Synthetic beta-amyloid peptide DAEFRHDSGYEVHHQKLVFFAEDVGSNKGAIIGLMVGGVVIA (Aβ, Peptide Specialty Laboratories GmbH, Heidelberg, Germany) was dissolved in 10% NH_4_OH, aliquoted, and lyophilized. Stock concentrations of Aβ (100 µM) were prepared in MEM containing 100 U/mL of penicillin, 100 µg/mL of streptomycin, sodium pyruvate, and GlutaMax (serum-free MEM). Only freshly prepared Aβ solutions were used for cell treatment. GSK and Aβ were further diluted to the required concentration with serum-free MEM medium. An equivalent amount of DMSO was added to the control and Aβ samples in all experiments.

### 3.3. Flow Cytometry Analysis

Flow cytometry analysis was performed to determine the effect of oxidative stress in HMC3 cells caused by Aβ and the effect of inhibitors on the level of reactive oxygen species (ROS) and cell death by double staining with dihydrorhodamine 123 (DHR, Ex/Em = 507/525 nm, ThermoFisher Scientific, Waltham, MA, USA) and propidium iodide (PI, Ex/Em = 535/617 nm; Sigma, St. Louis, MO, USA), respectively. HMC3 cells were seeded in 12-well plates and cultured for 24 h at 37 °C. Then, the cells were treated with Aβ (10 µM) in the absence and presence of inhibitors (25 µM) for 24 h followed by incubation with DHR dye for 15 min at 37 °C in darkness. Stained cells were first washed with Versene solution (Gibco, ThermoFisher Scientific, Waltham, MA, USA) and detached by TrypLE Express (Gibco, ThermoFisher Scientific, Waltham, MA, USA). MEM medium was added to all samples to inactivate the TrypLE enzyme. Then, cells were incubated with 10 μg/mL of PI for 1 min before analysis in a BD LSR Fortessa flow cytometer (Becton Dickinson, Franklin Lakes, NJ, USA), and data analysis was performed using FlowJo software, version 10.8.1 (Tree star, Ashland, OR, USA). The experiments were repeated triplicate and were expressed as mean ± SD.

### 3.4. Statistical Analysis

The data are shown as mean ± standard deviation measure from triplicate values obtained from 3 independent experiments. *p* values were determined by a two-way ANOVA or one-way ANOVA as indicated in the figure legends. *p* ≤ 0.05 (*), *p* ≤ 0.01 (**), *p* ≤ 0.001 (***) were considered significant. GraphPad Prism software, version 7.01 (GraphPad Software, San Diego, CA, USA) was used to generate figures and perform statistical analysis.

### 3.5. Synthesis

Procedures for chemical synthesis, characterization of the compounds, and NMR spectra profiles are summarized in the electronic Supplementary Information File.

### 3.6. Molecular Docking Analysis

The original structure of the human NADPH oxidase 2 was taken from the PDB: NOX2 (8wej). For docking, we took chain B of the 8wej structure and removed all the low-molecular-weight ligands and water molecules.

Molecular modeling of NOX2 complexes with inhibitors (Table 1) and docking were performed using the Chemical Computing Group’s Molecular Operating Environment (MOE) software (ver. 2019.0102) both to NADPH binding site and to a whole protein in blind mode. Ligand conformations were generated with the bond rotation method and then placed in the site with the Triangle Matcher method and ranked with the London dG scoring function. For each ligand, a docking pose with the best affinity was obtained. The docking structure of NADPH to NOX2 was used as a control (Table 1). Binding affinity of the inhibitors and NADPH to NOX2 was calculated by London dG scoring function and presented as Gibbs energy change (ΔG).

### 3.7. MTT Cytotoxicity Assay

To evaluate the cytotoxicity of NOX2 inhibitors, the commercially available GSK2795039 (GSKs, MedChemExpress, NJ, USA) and synthesized GSK2795039, NCATS-SM7270, or IMBIOC-1 were added to HMC3 cells at concentrations ranging from 0 to 100 µM and incubated for 24 h. The cytotoxicity of inhibitors was studied by the 3-(4,5-dimethylthiazol -2-yl)-2, 5-diphenyltetrazolium bromide (CyQUANT™ MTT Cell Proliferation Assay Kit, Sigma-Aldrich, St. Louis, MO, USA)) according to the manufacturer’s protocol. The absorbance was measured at 570 nm using Spark Multimode Microplate Reader (Tecan Group Ltd., Männedorf, Switzerland). Respiratory activity of untreated cells was taken as 100%. The experiment was performed in triplicate, and data were presented as mean ± SD.

### 3.8. Determination of Half Maximal Inhibitory Concentration (IC50)

HMC3 cells were treated with Aβ (10 µM) and NOX2 inhibitors for 24 h. Dynamics of ROS production were measured by 0.3 mM Amplex Red Reagent (Ex./Em. 560/590 nm, ThermoFisher Scientific, Waltham, MA, USA) for 30 min at 37 °C using a multifunctional plate reader CLARIOstar Plus (BMGlabtech, Ortenberg, Germany).

## 4. Conclusions

The structural complexity and high cost of the synthesis often limits novel promising drug candidates for research and commercial implementation for further treatment and studies of various pathologies. In this research, we proposed an optimized approach for the synthesis of NOX2 inhibitors, which can be scaled to obtain gram quantities of these substances. In the course of the synthesis development, we designed and obtained a new inhibitor IMBIOC-1, which is a positional isomer of the commercially available GSK2795039. IMBIOC-1 provides a simpler synthetic route from accessible starting reagents than GSK2795039, and its effectiveness in protecting microglial cells from Aβ-induced oxidative stress is even higher than that of the parent compound. Optimized conditions for the synthesis of 3-(indolin-6-yl)-4-(*N*-pyrazole-sulfonamide)-1*H*-pyrrolo[2,3-*b*]pyridine structures using standard building blocks allow us to expand the range of NOX2 inhibitors and promote these compounds for in vivo practical testing for their protective effect in various pathologies associated with inflammation in the brain, including AD, PD, traumatic brain injury, and others.

## Figures and Tables

**Table 1:** Interactions of NOX2 amino acid residues with the inhibitors *.

Compound	ΔG, kcal/mol	Hydrophobic Interactions	Hydrogen Bonds	π-Stacking	Salt Bridges
GSK2795039	−8.75	98Leu 324Tyr 339Pro	73Arg 97Asn 324Tyr	202Phe	–
NCATS-SM7270	−8.35	98Leu 202Phe 324Tyr 339Pro	73Arg 97Asn 324Tyr	–	–
IMBIOC-1	−8.85	76Leu 95Asp 98Leu 324Tyr 393Thr 570Phe	73Arg 97Asn 324Tyr 569Asn	–	–
NADPH	−11.89	–	203Glu 341Thr 354His 358Val	570Phe	338His

* For each residue, the type of interaction is indicated. The best binding affinity of inhibitors and NADPH to NOX2 is presented as ΔG (kcal/mol).

## Data Availability

The original contributions presented in this study are included in the article/Supplementary Materials. Further inquiries can be directed to the corresponding author(s).

## Supplementary Materials

The following supporting information can be downloaded at https://www.mdpi.com/article/10.3390/ijms26083647/s1.

## References

[1] McBean G.J., López M.G., Wallner F.K. (2017). Redox-based therapeutics in neurodegenerative disease. Br. J. Pharmacol..

[2] Ma M.W., Wang J., Zhang Q. (2017). NADPH oxidase in brain injury and neurodegenerative disorders. Mol. Neurodegener..

[3] Mason H., Rai G., Kozyr A., De Jonge N., Gliniewicz E., Berg L.J., Wald G., Dorrier C., Henderson M.J., Zakharov A. (2023). Development of an improved and specific inhibitor of NADPH oxidase 2 to treat traumatic brain injury. Redox Biol..

[4] Dohi K., Ohtaki H., Nakamachi T. (2010). Gp91phox (NOX2) in classically activated microglia exacerbates traumatic brain injury. J Neuroinflamm..

[5] Surace M.J., Block M.L. (2012). Targeting microglia-mediated neurotoxicity: The potential of NOX2 inhibitors. Cell. Mol. Life Sci..

[6] Park L., Zhou P., Pitstick R., Capone C., Anrather J., Norris E.H., Younkin L., Younkin S., Carlson G., McEwen B.S. (2008). Nox2-derived radicals contribute to neurovascular and behavioral dysfunction in mice overexpressing the amyloid precursor protein. Proc. Natl. Acad. Sci. USA.

[7] Belarbi K., Cuvelier E., Destée A. (2017). NADPH oxidases in Parkinson’s disease: A systematic review. Mol. Neurodegener..

[8] Reis J., Massari M., Marchese S., Ceccon M., Aalbers F.S., Corana F., Valente S., Mai A., Magnani F., Mattevi A. (2020). A closer look into NADPH oxidase inhibitors: Validation and insight into their mechanism of action. Redox Biol..

[9] Liu F.-C., Yu H.-P., Chen P.-J., Yang H.-W., Chang S.-H., Tzeng C.-C., Cheng W.-J., Chen Y.-R., Chen Y.-L., Hwang T.-L. (2019). A novel NOX2 inhibitor attenuates human neutrophil oxidative stress and ameliorates inflammatory arthritis in mice. Redox Biol..

[10] Juric M., Rawat V., Amaradhi R., Zielonka J., Ganesh T. (2023). Novel NADPH Oxidase-2 Inhibitors as Potential Anti-Inflammatory and Neuroprotective Agents. Antioxidants.

[11] Sorce S., Stocker R., Seredenina T., Holmdahl R., Aguzzi A., Chio A., Depaulis A., Heitz F., Olofsson P., Olsson T. (2017). NADPH oxidases as drug targets and biomarkers in neurodegenerative diseases: What is the evidence?. Free Radic. Biol. Med..

[12] Hirano K., Chen W.S., Chueng A.L., Dunne A.A., Seredenina T., Filippova A., Ramachandran S., Bridges A., Chaudry L., Pettman G. (2015). Discovery of GSK2795039, a Novel Small Molecule NADPH Oxidase 2 Inhibitor. Antioxid. Redox Signal..

[13] Padilha E.C., Shah P., Rai G., Xu X. (2021). NOX2 inhibitor GSK2795039 metabolite identification towards drug optimization. J. Pharm. Biomed. Anal..

[14] Deng H., Zhang Y., Li G.-G., Yu H.-H., Bai S., Guo G.-Y., Guo W.-L., Ma Y., Wang J.-H., Liu N. (2021). P2 × 7 receptor activation aggravates NADPH oxidase 2-induced oxidative stress after intracerebral hemorrhage. Neural Regener. Res..

[15] Wang M., Luo L. (2020). An Effective NADPH Oxidase 2 Inhibitor Provides Neuroprotection and Improves Functional Outcomes in Animal Model of Traumatic Brain Injury. Neurochem. Res..

[16] Malkov A., Popova I., Ivanov A., Jang S.S., Yoon S.Y., Osypov A., Huang Y., Zilberter Y., Zilberter M.A. (2021). β initiates brain hypometabolism, network dysfunction and behavioral abnormalities via NOX2-induced oxidative stress in mice. Commun. Biol..

[17] Chen D.W., Duncan S., King N.P., Lee K.C., Mak S.Y., Rivers D.A. (2012). Novel compounds.

[18] Gribble G.W., Nutaitis C.F. (1987). Reactions of Sodium Borohydride in Acidic Media; XVI.1 N-Methylation of Amines with Paraformaldehyde/Trifluoroacetic Acid. Synthesis.

[19] Poot A.J., van Ameijde J., Slijper M., van den Berg A., Hilhorst R., Ruijtenbeek R., Rijkers D.T.S., Liskamp R.M.J. (2009). Development of Selective Bisubstrate-Based Inhibitors Against Protein Kinase C (PKC) Isozymes By Using Dynamic Peptide Microarrays. ChemBioChem.

[20] Liu X., Zhang Y., Huang W., Tan W., Zhang A. (2018). Design. Synthesis and pharmacological evaluation of new acyl sulfonamides as potent and selective Bcl-2 inhibitors. Bioorg. Med. Chem..

[21] Liu X., Shi Y., Liu R., Song K., Chen L. (2024). Structure of human phagocyte NADPH oxidase in the activated state. Nature.

[22] Heneka M.T., Carson M.J., El Khoury J., Landreth G.E., Brosseron F., Feinstein D.L., Jacobs A.H., Wyss-Coray T., Vitorica J., Ransohoff R.M. (2015). Neuroinflammation in Alzheimer’s disease. Lancet Neurol..

[23] Maurya S.K., Bhattacharya N., Mishra S., Bhattacharya A., Banerjee P., Senapati S., Mishra R. (2021). Microglia Specific Drug Targeting Using Natural Products for the Regulation of Redox Imbalance in Neurodegeneration. Front. Pharmacol..

[24] Sarlus H., Heneka M.T. (2017). Microglia in Alzheimer’s disease. J. Clin. Investig..

