# Supplementary material for: Improved Synthesis of Effective 3-(Indolin-6-yl)-4-(N-pyrazole-sulfonamide)-1H-pyrrolo[2,3-b]pyridine-Based Inhibitors of NADPH Oxidase 2
Source: Int J Mol Sci. 2025 Apr 12;26(8):3647. doi: 10.3390/ijms26083647 (PMC12026636; doi:10.3390/ijms26083647)
Supplement: Supplementary file 1 [file ijms-26-03647-s001.zip › ijms-3516003-supplementary.pdf]

ELECTRONIC SUPPLEMENTARY MATERIAL

Improved Synthesis of Effective 3-(Indolin-6-yl)-4-(*N*-pyrazole-sulfonamide)-1*H*-pyrrolo[2,3-*b*]pyridine-Based Inhibitors of NADPH Oxidase 2

Konstantin V. Potapov [1,2,†], Dmitry N. Platonov [2,†], Aleksandr Yu. Belyy [2], Maxim A. Novikov [1,2], Yury V. Tomilov [2], Anastasia A. Anashkina [1], Kristina A. Mukhina [1], Olga I. Kechko [1], Pavel N. Solyev [1,*], Roman A. Novikov [1,2,*], Alexander A. Makarov [1], and Vladimir A. Mitkevich [1]

[1] Engelhardt Institute of Molecular Biology of the Russian Academy of Sciences, 119991 Moscow, Russia;
[2] Zelinsky Institute of Organic Chemistry of the Russian Academy of Sciences, 119991 Moscow, Russia.
* Correspondence: Pavel Solyev, e-mail solyev@gmail.com; Roman Novikov, novikovfff@bk.ru.
† These authors contributed equally to this work.

Chemical synthesis: Materials and methods ---------- S2
6-Bromo-1-methylindoline (**1**) ---------- S2
6-Bromo-1-methyl-1H-indole (**2**) ---------- S2
General procedure for the Miyaura borylation. ---------- S3
1-Methyl-6-(4,4,5,5-tetramethyl-1,3,2-dioxaborolan-2-yl)indoline (**3**) ---------- S3
1-Methyl-6-(4,4,5,5-tetramethyl-1,3,2-dioxaborolan-2-yl)-1H-indole (**4**) ---------- S3
1*H*-Pyrrolo[2,3-*b*]pyridine 7-oxide (**5**) ---------- S3
4-Bromo-1*H*-pyrrolo[2,3-*b*]pyridine (**6**) ---------- S4
4-Bromo-3-iodo-1*H*-pyrrolo[2,3-*b*]pyridine (**7**) ---------- S4
4-Bromo-3-iodo-1-isopropyl-1*H*-pyrrolo[2,3-*b*]pyridine (**8**) ---------- S4
4-Bromo-3-iodo-1-cyclopropyl-1*H*-pyrrolo[2,3-*b*]pyridine (**9**) ---------- S5
1-Methyl-1*H*-pyrazole-4-sulfonyl chloride (**10**) ---------- S5
1-Methyl-1*H*-pyrazole-4-sulfonamide (**11**) ---------- S5
1-Methyl-1*H*-pyrazole-3-sulfonamide (**13**) ---------- S6
General procedure for the Suzuki coupling ---------- S6
4-Bromo-1-isopropyl-3-(1-methylindolin-6-yl)-1*H*-pyrrolo[2,3-*b*]pyridine (**14**) ---------- S6
4-Bromo-1-cyclopropyl-3-(1-methyl-1*H*-indol-6-yl)-1*H*-pyrrolo[2,3-*b*]pyridine (**15**) ---------- S6
General procedure for the Buchwald–Hartwig reaction ---------- S7
N-(1-Isopropyl-3-(1-methylindolin-6-yl)-1*H*-pyrrolo[2,3-*b*]pyridin-4-yl)-1-methyl-1*H*-pyrazole-3-sulfonamide (**16**) ---------- S7
N-(1-Isopropyl-3-(1-methylindolin-6-yl)-1H-pyrrolo[2,3-*b*]pyridin-4-yl)-1-methyl-1*H*-pyrazole-4-sulfonamide (**17**) ---------- S7
N-(1-Cyclopropyl-3-(1-methyl-1*H*-indol-6-yl)-1*H*-pyrrolo[2,3-*b*]pyridin-4-yl)-1-methyl-1*H*-pyrazole-3-sulfonamide (**18**) ---------- S8
NMR spectra of the products ---------- S9-S35
HPLC chromatograms and HRMS spectra of the **16** (GSK2795039), **17** (IMBIOC-1) and **18** (NCATS-SM7270) ---------- S35-S38

Chemical synthesis.

Materials and Methods

All reagents and catalysts were purchased from Sigma-Aldrich, Acros, J&K Scientific and TCI Europe and used without further purification unless otherwise mentioned. TLC analysis was performed on Silufol chromatographic plates. For preparative chromatography, silica gel 60 (0.040–0.063 mm) was used. ^{1}H, ^{13}C NMR spectra were recorded on a Bruker AVANCE II 300 MHz (300.1, 75.5 MHz and 282.4 MHz, respectively) and a Bruker AMX III 400 MHz (400.1, 100.6 MHz and 376.5 MHz, respectively) spectrometers in $CDCl_3$, containing 0.05% Me_4Si as the internal standard. Determination and verification of the structures of obtained compounds and assignments of ^{1}H and ^{13}C signals were made using 1D and 2D DEPT, COSY, HSQC and HMBC spectra. High-resolution mass spectra were recorded on a Bruker Daltonics micrOTOF-Q II device (electrospray ionization). Measurements were carried out in positive ion mode. Samples were injected into the spray chamber of the mass spectrometer from an Agilent 1260 liquid chromatograph equipped with an Agilent Poroshell 120 EC-C18 column (3.0 × 50 mm; 2.7 μm); the flow rate was 0.4 mL min^{-1}; the samples of compounds were loaded using autosampler from acetonitrile solution and eluted in the following gradient of acetonitrile (A) in water: 0–6 min—0%–85% A, 6–7.5 min—85% A, 7.5–8 min—85%–0% A, 8–10 min—0% A. Preparative HPLC were performed on Thermo-Finnigan Surveyor equipped with UV-VIS detector on Supelco Ascentis C8 5 μm 250 mm × 10 mm chromatographic column.

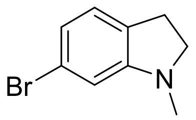

6-Bromo-1-methylindoline (1)

Paraformaldehyde (600 mg, 20.00 mmol) and $NaBH_3CN$ (314 mg, 10.00 mmol) were added to a stirred solution of 6-bromoindole (392 mg, 2.00 mmol) in 10 ml of glacial acetic acid. The mixture was stirred for 3h at room temperature, quenched with aq. NaOH solution and extracted with CH_2Cl_2. The combined organic phases were washed with water and brine, dried over $MgSO_4$, and concentrated *in vacuo*. The crude product was purified by silica gel column chromatography (eluent: petroleum ether−AcOEt, 10:1) to afford the target compounds as pale-yellow oil. Yield: 401 mg (95%).

^{1}H NMR (300 MHz, $CDCl_3$), δ: 6.88 (dt, *J* = 7.7, 1.2 Hz, 1H), 6.74 (dd, *J* = 7.7, 1.7 Hz, 1H), 6.54 (d, *J* = 1.7 Hz, 1H), 3.32 (t, *J* = 8.2 Hz, 2H), 2.87 (t, *J* = 8.2 Hz, 1H), 2.72 (s, 3H). ^{13}C NMR (75 MHz, $CDCl_3$), δ: 154.8, 129.3, 125.3, 121.1, 120.1, 110.0, 56.1, 35.7, 28.2.

HRMS (ESI) of $C_9H_{10}BrN$, *m/z*: calcd for $[M+Na]^+$ 233.9889 (^{79}Br), 235.9869 (^{81}Br); found 233.9885 and 235.9866.

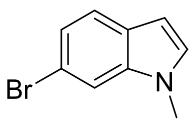

6-Bromo-1-methyl-1*H*-indole (2)

6-Bromoindole (500 mg, 2.55 mmol) was added to the stirred suspension of NaH (60% dispersion in oil, 204 mg, 5.1 mmol) in dry DMF (4 mL). The mixture was stirred for 1 h followed by the addition of MeI (724 mg, 5.1 mmol). The solution was stirred at room temperature overnight. The mixture was quenched with H_2O and extracted with EtOAc. The combined organic phases were washed with water and brine, dried over $MgSO_4$, and concentrated *in vacuo*. The crude product was purified by silica gel column chromatography (eluent: petroleum ether−AcOEt, 50:1) to afford the target compounds as pale-yellow oil. Yield: 525 mg (98%).

^1H NMR (300 MHz, $CDCl_3$), δ: 7.50 – 7.40 (m, 2H), 7.23 – 7.14 (m, 1H), 6.99 (d, J = 3.1 Hz, 1H), 6.43 (dd, J = 3.1, 0.9 Hz, 1H), 3.71 (s, 3H). ^{13}C NMR (75 MHz, $CDCl_3$), δ: 137.6, 129.5, 127.3, 122.6, 122.1, 115.2, 112.3, 101.3, 32.9.

HRMS (ESI) of C_9H_8BrN, *m/z*: calcd for $[M+H]^+$ 209.9913 (^{79}Br), 211.9893 (^{81}Br), found 209.9915 and 211.9895.

General procedure for the Miyaura borylation

B_2pin_2 (528 mg, 2.08 mmol) was added to the solution of the Br-containing compound (1.89 mmol) in 20 ml of dry THF under Ar atmosphere. Then, AcOK (556 mg, 5.67 mmol), XPhos (36 mg, 0.08 mmol), and (2-methylallyl)palladium(II) chloride dimer (8 mg, 0.04 mmol) were added to the solution, and the reaction mixture was stirred for 6 h at 80°C. The mixture was cooled down to room temperature, quenched with an aqueous solution of NH_4Cl and extracted with Et_2O. The combined organic phases were washed with water and brine, dried over $MgSO_4$, and concentrated in vacuo. The crude product was purified by silica gel column chromatography.

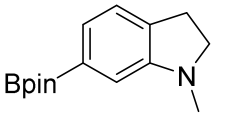

1-Methyl-6-(4,4,5,5-tetramethyl-1,3,2-dioxaborolan-2-yl)indoline (3)

The product was purified by silica gel column chromatography using the eluting system petroleum ether−AcOEt, 10:1 to afford the target compound as yellow solid. Yield: 411mg (84%).

^1H NMR (300 MHz, $CDCl_3$), δ: 7.18 (d, J = 7.2 Hz, 1H), 7.08 (d, J = 7.2 Hz, 1H), 6.90 (s, 1H), 3.25 (t, J = 8.2 Hz, 2H), 2.92 (t, J = 8.1 Hz, 2H), 2.77 (s, 3H), 1.32 (s, 12H). ^{13}C NMR (75 MHz, $CDCl_3$), δ: 152.9, 134.1, 125.2, 123.8, 112.5, 83.5, 56.0, 36.3, 29.0, 24.8.

HRMS (ESI) of $C_{15}H_{22}BNO_2$, *m/z*: calcd for $[M+H]^+$ 260.1819, found 260.1822.

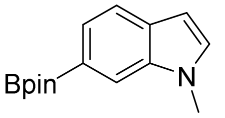

1-Methyl-6-(4,4,5,5-tetramethyl-1,3,2-dioxaborolan-2-yl)-1*H*-indole (4)

The product was purified by silica gel column chromatography using the eluting system petroleum ether−AcOEt, 7:1 to afford the target compound as yellow solid. Yield: 432 mg (89%).

^1H NMR (300 MHz, $CDCl_3$), δ: 7.83 (d, J = 1.2 Hz, 1H), 7.62 (dd, J = 7.9, 0.8 Hz, 1H), 7.59 – 7.52 (m, 1H), 7.09 (d, J = 3.1 Hz, 1H), 6.47 (dd, J = 3.1, 0.9 Hz, 1H), 3.81 (s, 3H), 1.37 (s, 12H). ^{13}C NMR (75 MHz, $CDCl_3$), δ: 136.4, 131.0, 130.2, 125.1, 120.1, 116.1, 101.0, 83.5, 32.9, 25.0, 24.9.

HRMS (ESI) of $C_{15}H_{20}BNO_2$, *m/z*: calcd for $[M+H]^+$ 258.1663, found 258.1667.

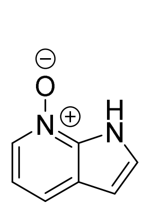

1*H*-Pyrrolo[2,3-*b*]pyridine 7-oxide (5)

To a solution of 7-azaindole (500 mg, 4.2 mmol) in dichloromethane (20 mL), *m*-chloroperbenzoic acid (77%, 1.42 g, 6.35 mmol) was added, and the reaction mixture was stirred for 3 h. The solvent was removed *in vacuo*, and the residue was purified by column chromatography (CH_2Cl_2/MeOH, 9:1), affording the *N*-oxide as a yellow solid. Yield: 550 mg (97%).

^{1}H NMR (400 MHz, $CDCl_3$), δ: 13.77 (br s, 1H), 8.27 (dd, 1H, *J* = 6.3, 0.9 Hz), 7.69 (dd, 1H, *J* = 7.7, 0.9 Hz), 7.43 (d, 1H, *J* = 3.3 Hz), 7.05 (dd, 1H, *J* = 7.7, 6.3 Hz), 6.54 (d, 1H, *J* = 3.3 Hz). ^{13}C NMR (100 MHz, $CDCl_3$), δ: 139.0, 131.6, 127.6, 125.6, 122.7, 115.6, 102.4.

HRMS (ESI) of $C_7H_6N_2O$, *m/z*: calcd for $[2M+H]^+$ 269.1033, found 269.1037.

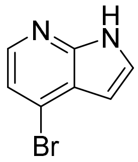

4-Bromo-1*H*-pyrrolo[2,3-*b*]pyridine (6)

1*H*-Pyrrolo[2,3-*b*]pyridine 7-oxide (3.0 g, 22.4 mmol) and tetramethylammonium bromide (4.13 g, 1.2 eq) were dissolved in DMF (30 ml). The Ms_2O (7.8 g, 2.0 eq) was added at 0°C in small portions. The mixture was stirred at 0°C for 1 h, at room temperature for 4 h, and was diluted with water (60 ml). The pH was adjusted to 7 with solid NaOH, and more than 130 ml of water was added. The resulting suspension was kept at 5°C for 1 h. The precipitate was separated by filtration, washed with ice-water, and dried over P_2O_5 in vacuum oven to give 4-bromo-1*H*-pyrrolo[2,3-b]pyridine. Yield: 2.47 g (56%).

^{1}H NMR (300 Hz, $CDCl_3$), δ: 10.77 (br, s, 1H), 8.14 (d, *J* = 5.2 Hz, 1H), 7.42 (s, 1H), 7.31 (d, *J* = 5.1 Hz, 1H), 6.57 (s, 1H). ^{13}C NMR (75 MHz, DMSO-d6) δ 148.2, 143.0, 127.2, 123.5, 120.6, 118.4, 99.4.

HRMS (ESI) of $C_7H_5BrN_2$, *m/z*: calcd for $[M+Na]^+$ 218.9528 (^{79}Br), 220.9508 (^{81}Br), found 218.9524 and 220.9511.

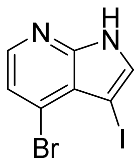

4-Bromo-3-iodo-1*H*-pyrrolo[2,3-*b*]pyridine (7)

4-Bromo-1*H*-pyrrolo[2,3-*b*]pyridine (0.713 g, 3.62 mmol) was dissolved in DCM (25 mL) under nitrogen gas and cooled to 0°C. *N*-Iodosuccinimide (0.896 g, 3.98 mmol) was added to the solution, and the reaction was stirred at 0°C for 1 hour before being quenched by the saturated Na_2SO_3 solution. The mixture was filtered *in vacuo* and washed with DCM. The filtrate was evaporated and dried under high vacuum to give the target compound as yellow solid. Yield: 1.01 g (86%).

^{1}H NMR (300 MHz, DMSO-d_6), δ: 12.41 (br. s, 1H), 8.03 (d, *J* = 5.1 Hz, 1H), 7.76 (br. s, 1H), 7.30 (d, *J* = 5.1 Hz, 1H). ^{13}C NMR (75 MHz, DMSO-d6) δ 147.5, 144.0, 128.0, 123.1, 120.6, 118.4, 51.6.

HRMS (ESI) of $C_7H_4BrIN_2$, *m/z*: calcd for $[M+H]^+$ 322.8675 (^{79}Br), 324.8655 (^{81}Br), found 322.8671 and 324.8653.

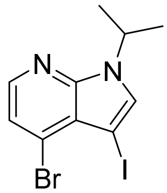

4-Bromo-3-iodo-1-isopropyl-1*H*-pyrrolo[2,3-*b*]pyridine (8)

Sodium hydride (0.13 g 60% in mineral oil, 3.25 mmol) was added portionwise to a cooled mixture of 4-bromo-3-iodo-1H-pyrrolo[2,3-*b*]pyridine (0.5 g, 1.54 mmol) in DMF (7 mL) at 0°C

and was stirred at that temperature for 15 min. 2-Iodopropane (0.34 g, 0.2 ml, 2.0 mmol) was then added, and the reaction mixture was allowed to warm to room temperature and stirred overnight. The reaction mixture was cooled to 0°C again and quenched with a saturated NH_4Cl solution (ca. 1 mL) and further diluted with water (ca. 20 mL). The aqueous layer was extracted with ethyl acetate (3×20 mL). The organic layer was dried and concentrated. The residue was purified on silica gel, eluting with ethyl acetate in cyclohexane (0-10%) to give the target compound. Yield: 500 mg (89%).

^{1}H NMR (300 MHz, $CDCl_3$), δ: 8.06 (d, *J* = 5.0 Hz, 1H), 6.49 (s, 1H), 7.26 (d, *J* = 5.0 Hz, 1H), 5.17 (q, *J* = 6.8 Hz, 1H), 1.50 (d, *J* = 6.8 Hz, 6H), 2.72 (s, 3H). ^{13}C NMR (75 MHz, $CDCl_3$), δ: 146.3, 143.1, 131.4, 125.8, 121.1, 119.0, 117.5, 46.6, 22.9. HRMS (ESI) of $C_{10}H_{10}BrIN_2$, m/z: calcd for $[M+H]^+$ 364.9145 and 366.9125; found 364.9139 and 366.9125.

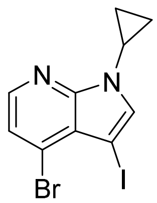

4-Bromo-3-iodo-1-cyclopropyl-1H-pyrrolo[2,3-*b*]pyridine (9)

To a suspension of cyclopropylboronic acid (265 mg, 3.0 mmol), 4-bromo-3-iodo-1*H*-pyrrolo[2,3-*b*]pyridine (500 mg, 1.54 mmol), and Na_2CO_3 (320 mg , 3 mmol) in 4 mL, dichloroethane was added to a suspension of $Cu(OAc)_2$ (281 mg, 1.54 mmol) and bipyridine (240 mg , 1.54 mmol) in hot DCE (1.0 mL). The mixture was heated to 60° and stirred for 10 h under air. The resulting mixture was cooled to room temperature, and a saturated aqueous NH_4Cl solution was added, followed by water. The organic layer was separated, and the aqueous layer was extracted with DCM (15 mL) for three times. The organic phase was dried with anhydride sodium sulphate and concentrated *in vacuo*. The residue was further purified by column chromatography on silica gel using DCM as eluent to afford the target compound. Yield: 220 mg (39%).

^{1}H NMR (300 MHz, $CDCl_3$), δ: 8.11 (d, *J* = 5.0 Hz, 1H), 7.38 (s, 1H), 7.27 (d, *J* = 5.1 Hz, 1H), 3.60 – 3.43 (m, 1H), 1.22 – 0.91 (m, 4H). ^{13}C NMR (75 MHz, $CDCl_3$), δ: 143.48, 134.82, 121.32, 50.35, 27.16, 6.45. HRMS (ESI) of $C_{10}H_8BrIN_2$, *m/z*: calcd for $[M+H]^+$ 362.8988 and 364.8968; found 362.8982 and 364.8968.

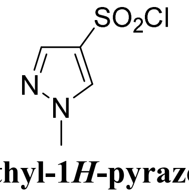

1-Methyl-1*H*-pyrazole-4-sulfonyl chloride (10)

N-Methylpyrazole (3.9 g, 47 mmol) was added dropwise to chlorosulfonic acid (16.42 g, 141 mmol) at 0°C, and, then, the reaction mixture was heated to 110°C and kept at this temperature for 4 hours. The reaction mass was cooled down and slowly poured onto ice. The product was filtered, washed with water, and dried *in vacuo* to afford the target compound as white powder. Yield: 3.82 g (45%).

^{1}H NMR (300 MHz, $CDCl_3$), δ: 8.05 (s, 1H), 7.99 (s, 1H), 4.03 (s, 3H). ^{13}C NMR (75 MHz, $CDCl_3$), δ: 138.6, 132.6, 126.7, 40.1.

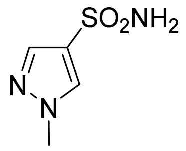

1-Methyl-1*H*-pyrazole-4-sulfonamide (11)

1-Methyl-1*H*-pyrazole-4-sulfonyl chloride (0.5 g, 2.77 mmol) was added to a stirred mixture of aq. ammonia (2 ml, 27.7 mmol) and THF (20 ml), and the solution was kept stirring for 30 minutes. Then, the solvents were evaporated from the mixture *in vacuo*. The product was extracted from the solid residue with ethyl acetate. The extract was evaporated *in vacuo* to give the target compound as yellowish crystals. Yield: 0.41 g (92%).

^{1}H NMR (300 MHz, DMSO-d$_6$), δ: 8.15 (s, 1H), 7.71 (s, 1H), 7.23 (s, 2H), 3.88 (s, 3H). ^{13}C NMR (75 MHz, DMSO-d$_6$), δ: 137.4, 131.6, 126.8, 39.3.

HRMS (ESI) of $C_4H_7N_3O_2S$, *m/z*: calcd for $[2M+H]^+$ 323.0591, found 323.0587.

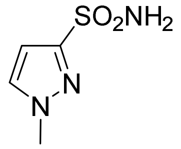

1-Methyl-1*H*-pyrazole-3-sulfonamide (13)

1-Methyl-1*H*-pyrazole-3-sulfonyl chloride (1.0 g., 5.5 mmol) was added to a stirred mixture of aq. ammonia (4 ml, 27.7 mmol.) and THF (40 ml), and the solution was kept stirring for 30 minutes. Then, the solvents were evaporated from the mixture *in vacuo*. The product was extracted from the solid residue with ethyl acetate. The extract was evaporated *in vacuo* to give the target product as yellowish crystals. Yield: 0.70 g (79%).

^{1}H NMR (300 MHz, DMSO-d$_6$), δ: 7.82 (d, J = 2.2 Hz, 1H), 7.36 (s, 2H), 6.56 (d, J = 2.2 Hz, 1H), 3.91 (s, 3H). ^{13}C NMR (75 MHz, DMSO-d$_6$), δ: 153.7, 132.9, 105.5, 39.6.

HRMS (ESI) of $C_4H_7N_3O_2S$, *m/z*: calcd for $[2M+H]^+$ 323.0591, found 323.0695.

General procedure for the Suzuki coupling

Bis(triphenylphosphine)palladium(II) dichloride (164 mg, 0.23 mmol) and Na_2CO_3 (2.52 g, 23.34 mmol) were added to the solution of the corresponding boronic ester (2.33 mmol) and azaindole (1.95 mmol) in DMF (4 ml), CH_3CN (4 ml), CH_2Cl_2 (2 ml), and H_2O (11 ml). The reaction mixture was stirred at 65°C for 2 h, cooled down to room temperature, quenched with water, and extracted with CH_2Cl_2. The combined organic phases were washed with water and brine, dried over $MgSO_4$, and concentrated *in vacuo*. The crude product was purified by silica gel column chromatography to afford the target compound.

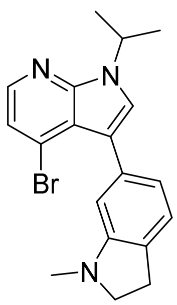

4-Bromo-1-isopropyl-3-(1-methylindolin-6-yl)-1*H*-pyrrolo[2,3-*b*]pyridine (14)

The product was obtained according to the general procedure for the Suzuki coupling and was purified by silica gel column chromatography using the eluting system petroleum ether−AcOEt, 4:1 to afford the target compound as yellow solid. Yield: 56%.

^{1}H NMR (300 MHz, $CDCl_3$), δ: 8.09 (d, *J* = 5.1 Hz, 1H), 7.34 (s, 1H), 7.26 (d, *J* = 5.0 Hz, 1H), 7.10 (dd, J = 7.4, 2.4 Hz, 1H), 6.80 (dd, *J* = 7.4, 1.5 Hz, 1H), 6.66 (dd, *J* = 4.7, 1.5 Hz, 1H), 5.24 (p, *J* = 6.8 Hz, 1H), 3.34 (td, *J* = 8.1, 6.3 Hz, 2H), 2.98 (dt, *J* = 10.5, 8.1 Hz, 2H), 1.54 (d, *J* = 6.8 Hz, 6H).

HRMS (ESI) of $C_{19}H_{20}BrN_3$, *m/z*: calcd for $[M+H]^+$ 370.0913 (^{79}Br), 372.0895 (^{81}Br), found 370.0917 and 372.0899.

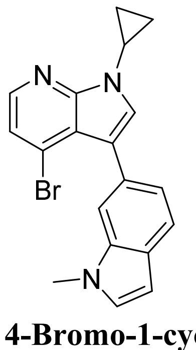

4-Bromo-1-cyclopropyl-3-(1-methyl-1*H*-indol-6-yl)-1*H*-pyrrolo[2,3-*b*]pyridine (15)

The product was obtained according to the general procedure for the Suzuki coupling and was purified by silica gel column chromatography using the eluting system petroleum ether−AcOEt, 3:1 to afford the target compound as yellow solid. Yield: 61%.

^{1}H NMR (300 MHz, $CDCl_3$), δ: 8.17 (d, *J* = 5.1 Hz, 1H), 7.93 (s, 1H), 7.61 (d, *J* = 8.1 Hz, 1H), 7.41 (s, 1H), 7.28 (d, *J* = 5.1 Hz, 1H), 7.26–7.20 (m, 2H), 7.05 (d, *J* = 3.1 Hz, 1H), 6.51 (dd, *J* = 3.1, 0.9 Hz, 1H), 3.79 (s, 3H), 3.69 – 3.53 (m, 1H), 1.42 – 1.02 (m, 4H). ^{13}C NMR (75 MHz, $CDCl_3$), δ: 149.2, 143.0, 136.4, 129.1, 128.0, 127.5, 127.1, 125.2, 123.2, 120.9, 119.7, 119.3, 117.6, 111.8, 100.9, 32.9, 26.9, 6.5.

HRMS (ESI) of $C_{19}H_{16}BrN_3$, *m/z*: calcd for $[M+H]^+$ 366.0600 (^{79}Br), 368.0582 (^{81}Br), found 366.0604 and 368.0588.

General procedure for the Buchwald–Hartwig reaction

$Pd_2(dba)_3$ (21 mg, 0.022 mmol), Xantphos (28 mg, 0.048 mmol), and Cs_2CO_3 (213 mg, 0.65 mmol) were added to the solution of the corresponding amide (0.42 mmol) and bromide (0.32 mmol) in 1,4-dioxane (3 ml). The reaction mixture was stirred at 115°C for 6 h, cooled down to room temperature, and, then, the solvents were evaporated from the mixture *in vacuo*. The crude product was purified by silica gel column chromatography to afford the target compound.

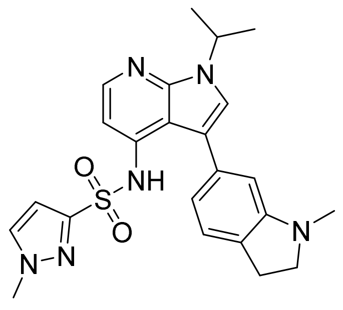

***N*-(1-Isopropyl-3-(1-methylindolin-6-yl)-1*H*-pyrrolo[2,3-*b*]pyridin-4-yl)-1-methyl-1*H*-pyrazole-3-sulfonamide (16)**

The product was obtained according to the general procedure for the Buchwald–Hartwig reaction and was purified by silica gel column chromatography using the eluting system petroleum ether−AcOEt, 1:4 to afford the target compound as brown solid. Yield: 76 mg (53%).

^{1}H NMR (300 MHz, $CDCl_3$), δ: 8.16 (d, *J* = 5.5 Hz, 1H), 7.34 (d, *J* = 2.3 Hz, 1H), 7.26 – 7.13 (m, 3H), 6.74 (d, *J* = 2.3 Hz, 1H), 6.72 (dd, *J* = 7.3, 1.6 Hz, 1H), 6.57 (d, *J* = 1.5 Hz, 1H), 5.18 (p, *J* = 6.7 Hz, 1H), 3.89 (s, 3H), 3.41 (t, *J* = 8.2 Hz, 2H), 3.01 (t, *J* = 8.6 Hz, 1H), 2.86 (s, 3H), 1.51 (d, *J* = 6.8 Hz, 6H). ^{13}C NMR (75 MHz, $CDCl_3$), δ: 154.56, 149.17, 148.09, 143.80, 138.27, 134.05, 131.75, 129.96, 124.98, 121.38, 118.23, 115.34, 108.39, 107.31, 102.91, 56.08, 45.48, 39.93, 35.90, 28.42, 22.88. HRMS (ESI) of $C_{23}H_{26}N_6O_2S$, *m/z*: calcd for $[M+H]^+$ 451.1911; found 451.1904.

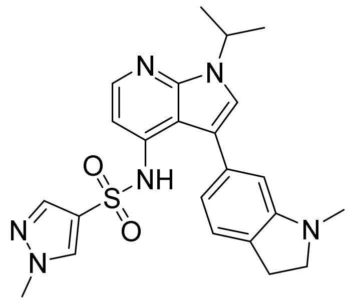

N-(1-Isopropyl-3-(1-methylindolin-6-yl)-1H-pyrrolo[2,3-*b*]pyridin-4-yl)-1-methyl-1*H*-pyrazole-4-sulfonamide (17)

The product was obtained according to the general procedure for the Buchwald–Hartwig reaction and was purified by silica gel column chromatography using the eluting system petroleum ether−AcOEt, 1:4 to afford the target compound as brown solid. Yield: 81 mg (56%).

^{1}H NMR (300 MHz, $CDCl_3$), δ: 8.21 (d, *J* = 5.4 Hz, 1H), 7.72 (s, 1H), 7.70 (s, 1H), 7.64 (d, *J* = 0.7 Hz, 1H), 7.24 – 7.11 (m, 3H), 6.60 (dd, *J* = 7.3, 1.6 Hz, 1H), 6.45 (d, *J* = 1.5 Hz, 1H), 5.19 (p, *J* = 6.8 Hz, 1H), 3.84 (s, 3H), 3.42 (t, *J* = 8.2 Hz, 2H), 3.03 (t, *J* = 8.2 Hz, 2H), 2.83 (s, 3H), 1.52 (d, *J* = 6.8 Hz, 6H). ^{13}C NMR (75 MHz, DMSO), δ: 154.15, 138.77, 134.39, 133.75, 130.54, 129.17, 127.31, 124.71, 123.66, 121.01, 118.57, 110.90, 108.33, 104.61, 104.37, 100.77, 56.17, 45.87, 36.17, 32.97, 28.36, 22.78. HRMS (ESI) of $C_{23}H_{26}N_6O_2S$, *m/z*: calcd for $[M+H]^+$ 451.1911; found 451.1898.

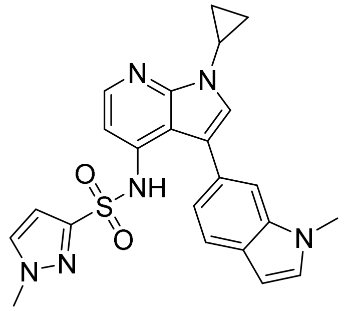

***N*-(1-Cyclopropyl-3-(1-methyl-1*H*-indol-6-yl)-1*H*-pyrrolo[2,3-*b*]pyridin-4-yl)-1-methyl-1*H*-pyrazole-3-sulfonamide (18)**

The product was obtained according to the general procedure for the Buchwald–Hartwig reaction and was purified by silica gel column chromatography using the eluting system petroleum ether−AcOEt, 1:5 to afford the target compound as brown solid. Yield: 60 mg (42%).

^{1}H NMR (300 MHz, $CDCl_3$), δ: 8.27 (d, *J* = 5.5 Hz, 1H), 7.87 (br.s, 1H), 7.76 (d, *J* = 8.1 Hz, 1H), 7.55 (s, 1H), 7.36 (d, *J* = 2.3 Hz, 1H), 7.30 (s, 1H), 7.20 (dd, *J* = 8.1, 1.5 Hz, 1H), 7.16 – 7.09 (m, 2H), 6.75 (d, *J* = 2.3 Hz, 1H), 6.55 (d, *J* = 3.1 Hz, 1H), 3.90 (s, 6H), 3.58 (tt, *J* = 7.1, 3.8 Hz, 1H), 1.23 – 1.01 (m, 4H). ^{13}C NMR (75 MHz, $CDCl_3$), δ: 149.97, 149.16, 144.35, 138.47, 137.27, 131.77, 129.86, 128.07, 127.48, 125.39, 122.00, 120.79, 115.33, 109.76, 108.57, 103.47, 101.14, 39.96, 33.09, 26.73, 6.42. HRMS (ESI) of $C_{23}H_{26}N_6O_2S$, *m/z*: calcd for $[M+H]^+$ 447.1598; found 447.1596.

^{1}H NMR (300 MHz) in $CDCl_3$ for **10**

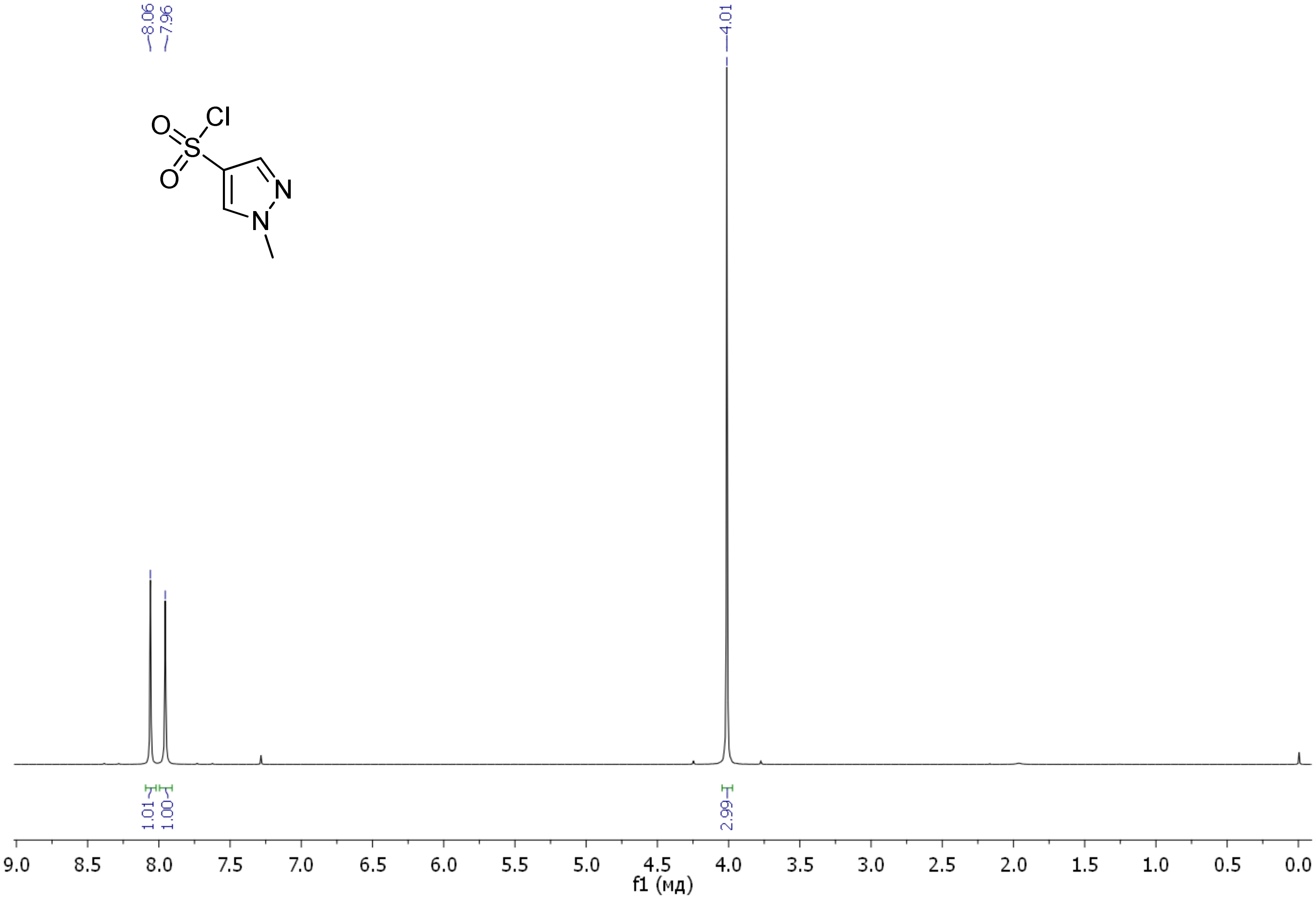

^{13}C NMR (75.5 MHz) in $CDCl_3$ for **10**

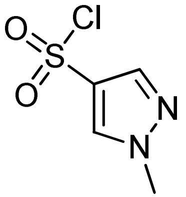

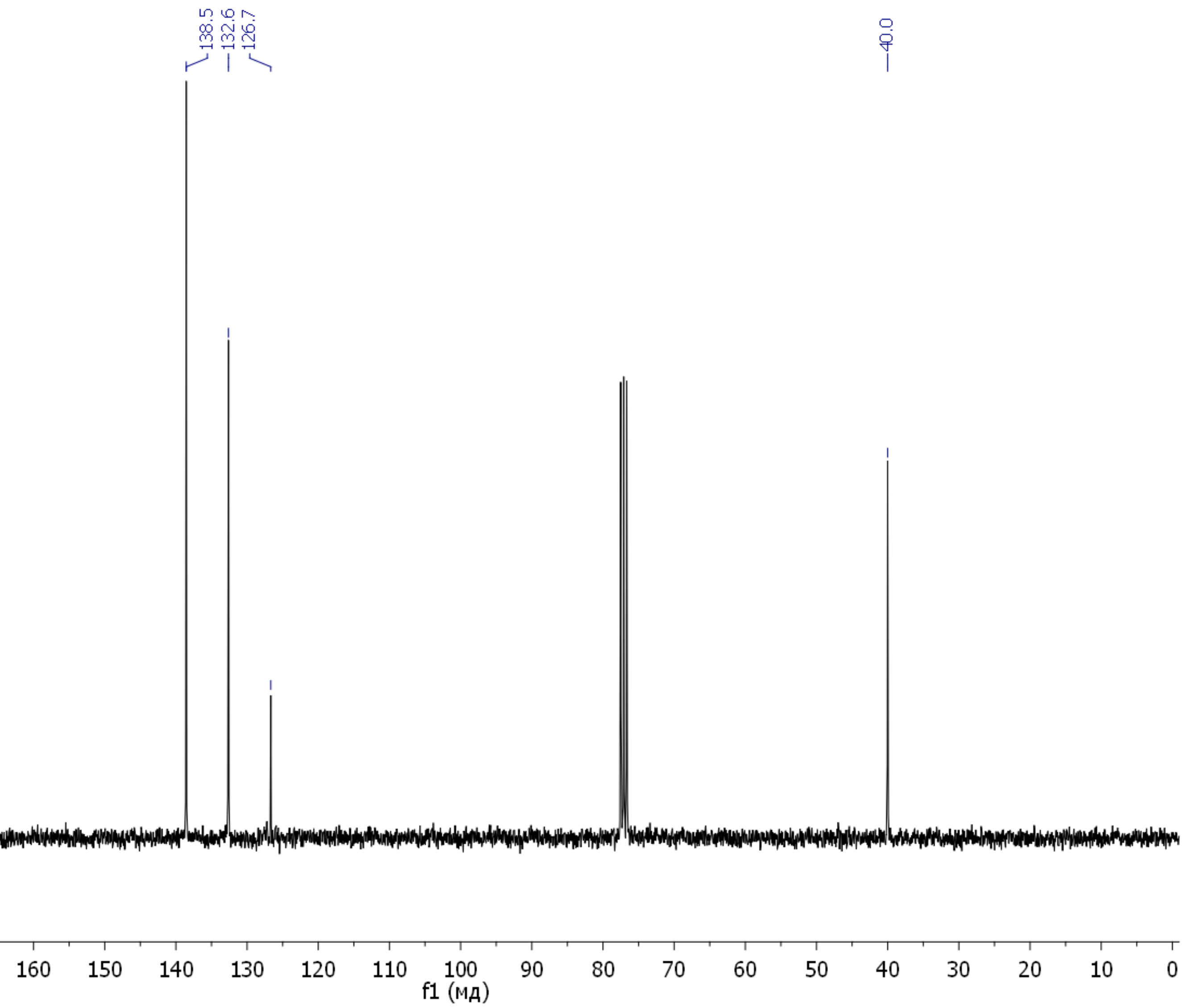

^{1}H NMR (300 MHz) in DMSO-d6 for **11**

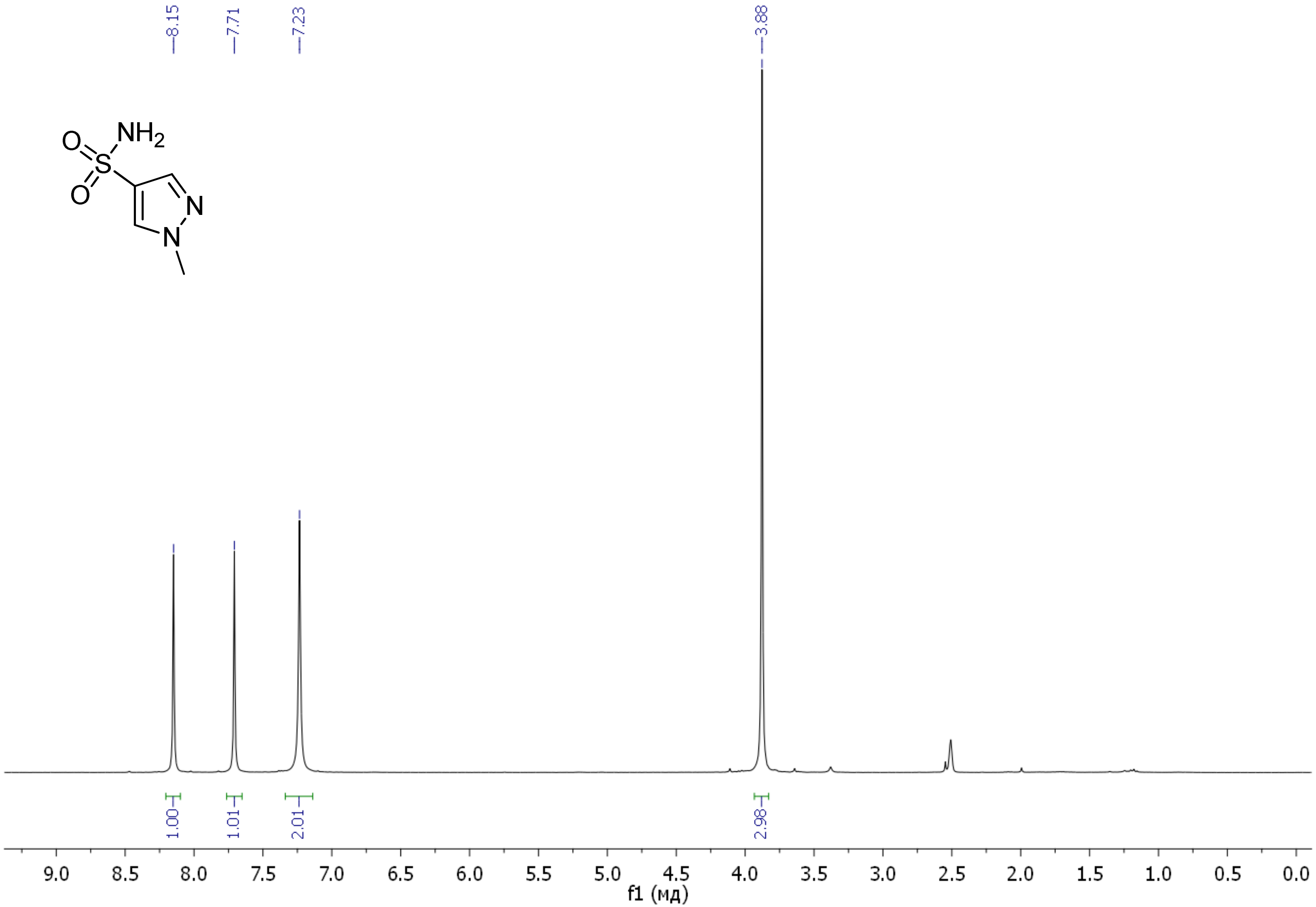

^{13}C NMR (75.5 MHz) in DMSO-d6 for **11**

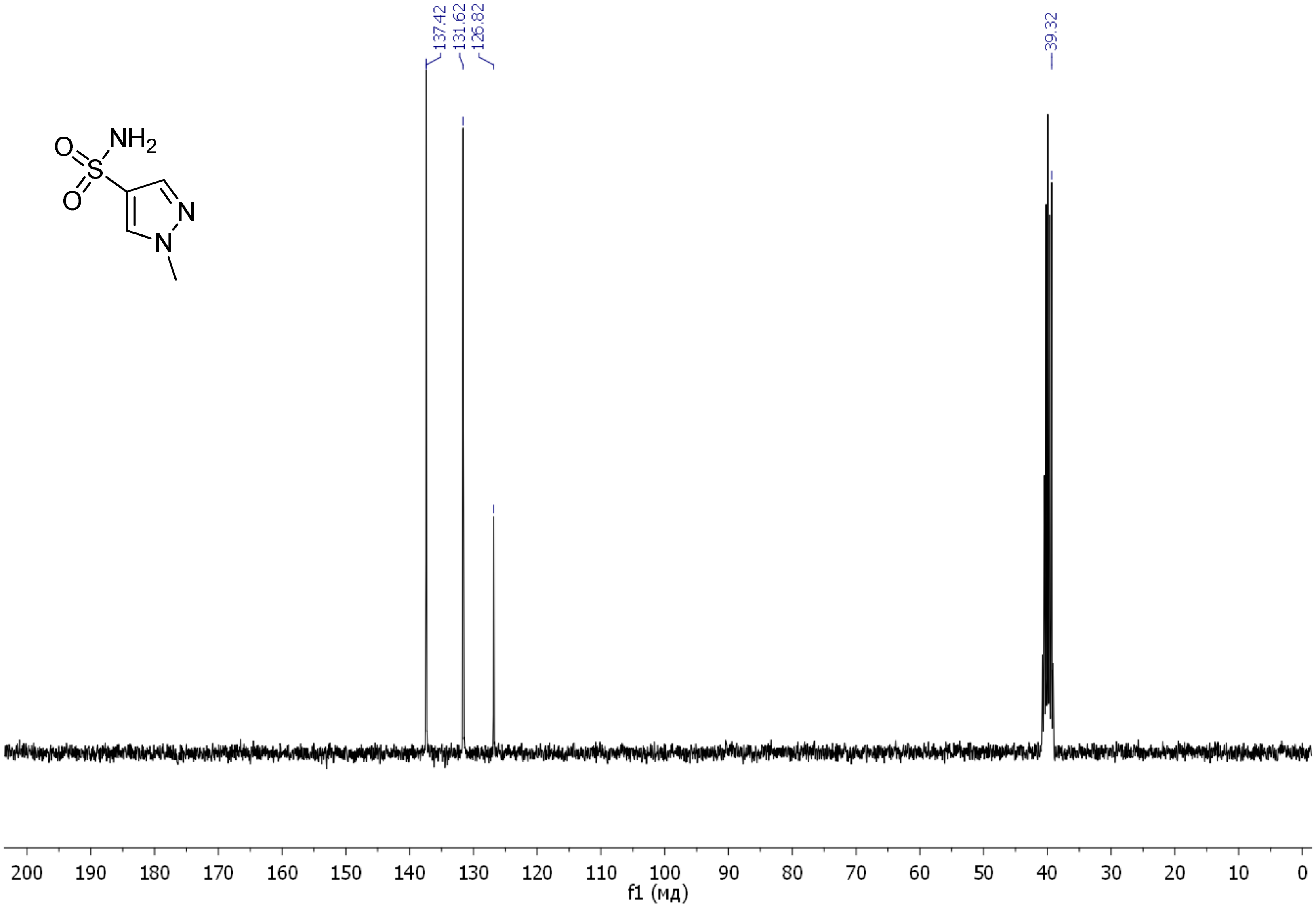

^{1}H NMR (300 MHz) in $CDCl_3$ for **13**

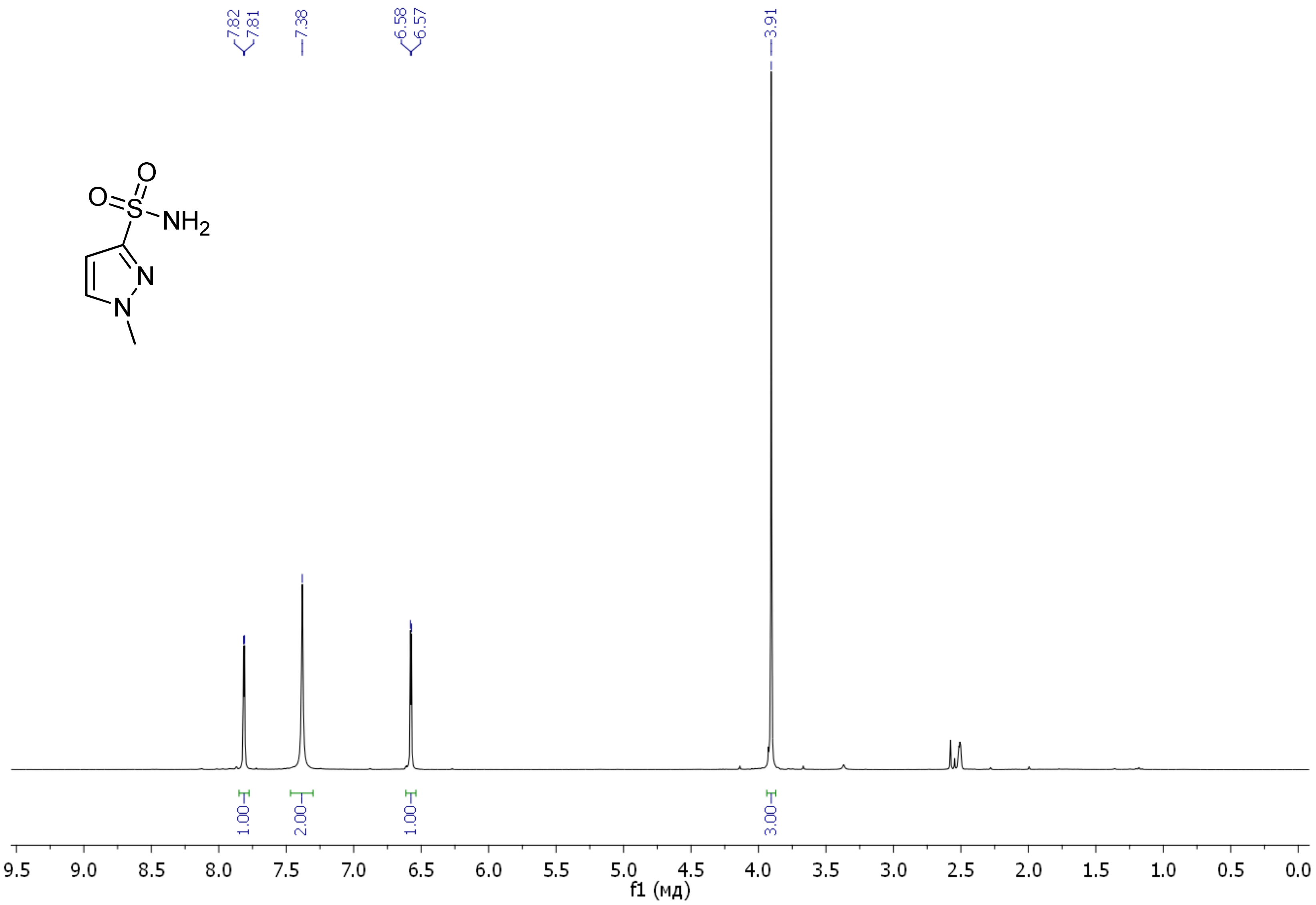

^{13}C NMR (75.5 MHz) in DMSO-d6 for **13**

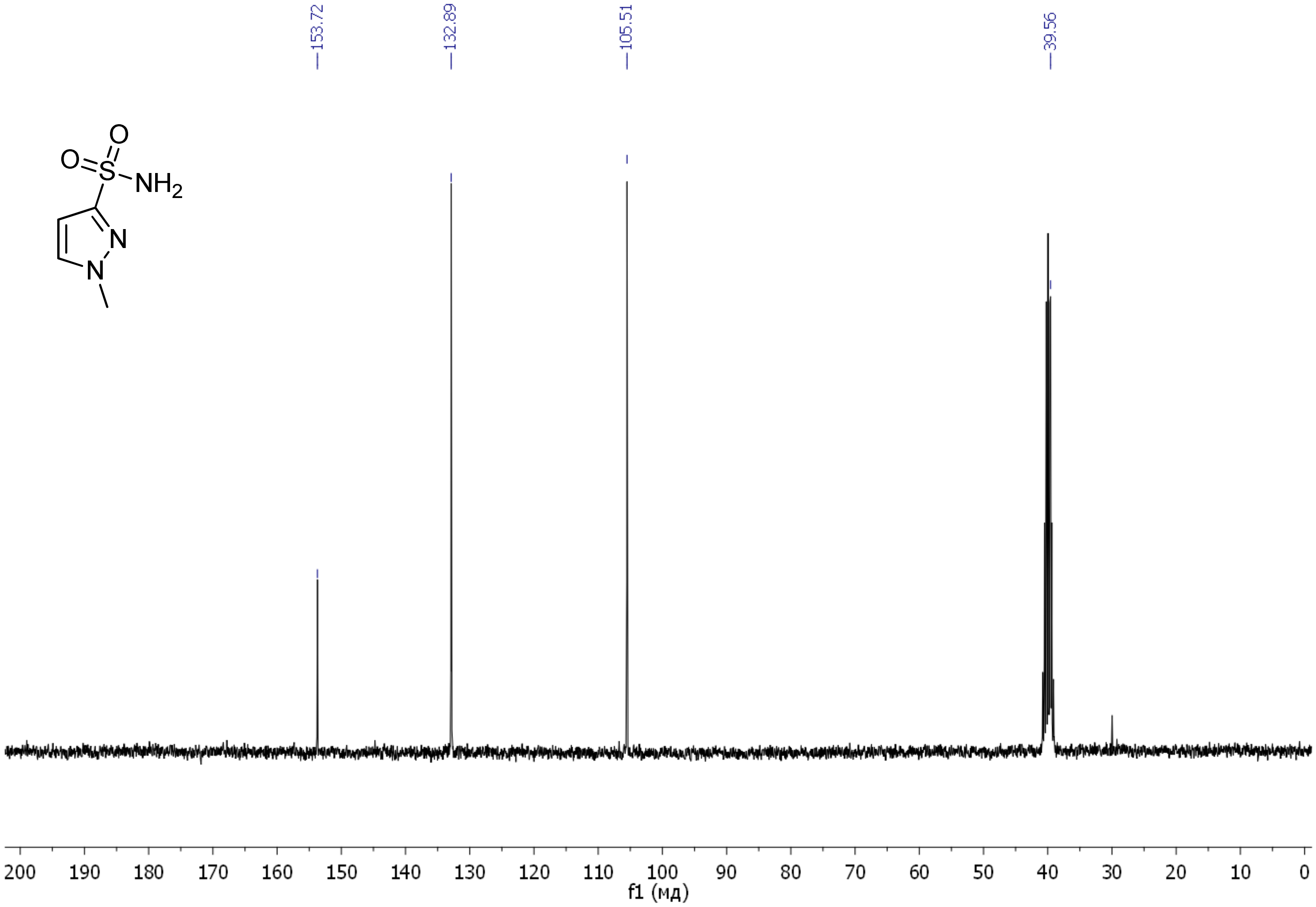

^{1}H NMR (300 MHz) in $CDCl_3$ for **1**

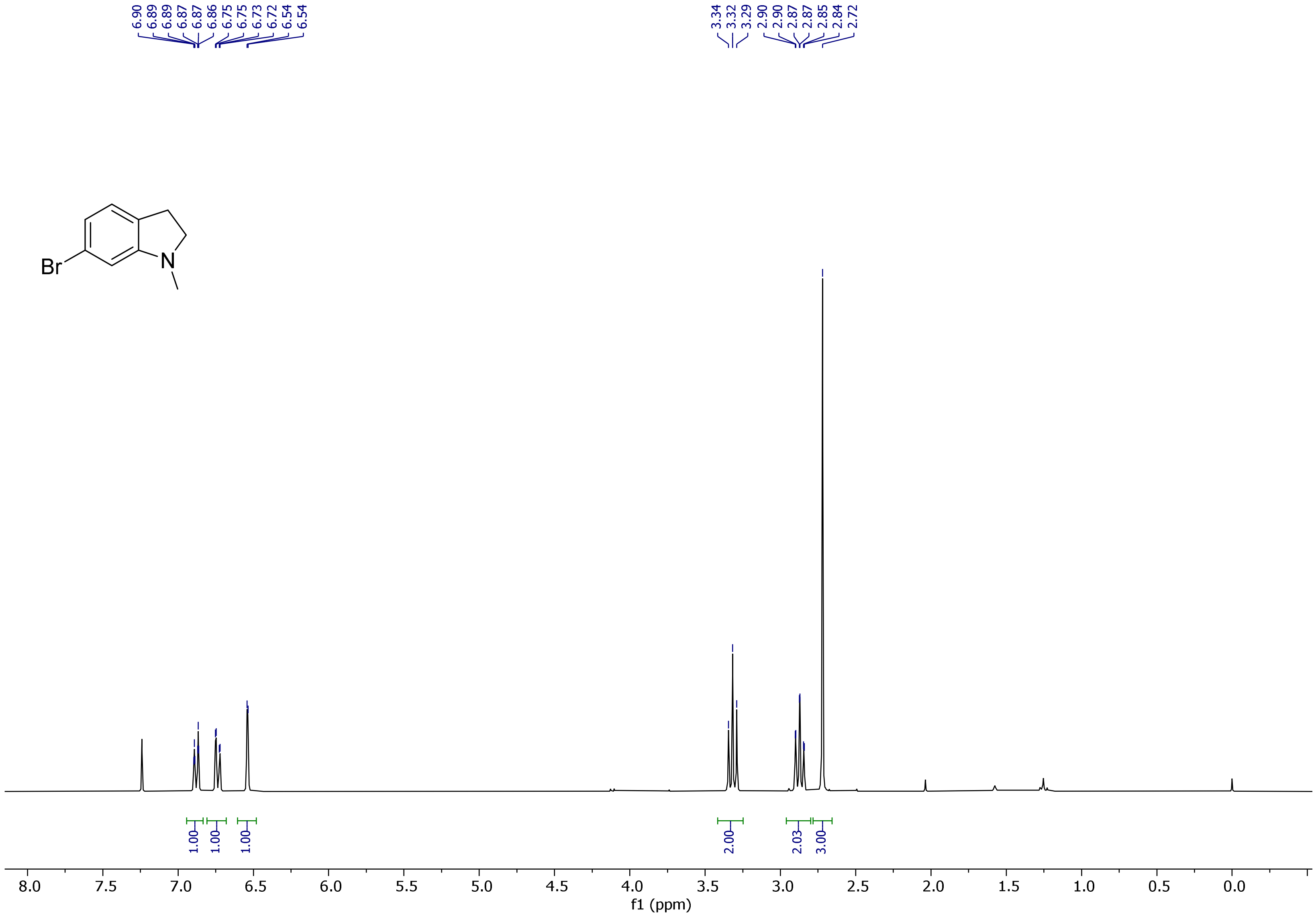

^{13}C NMR (75.5 MHz) in $CDCl_3$ for **1**

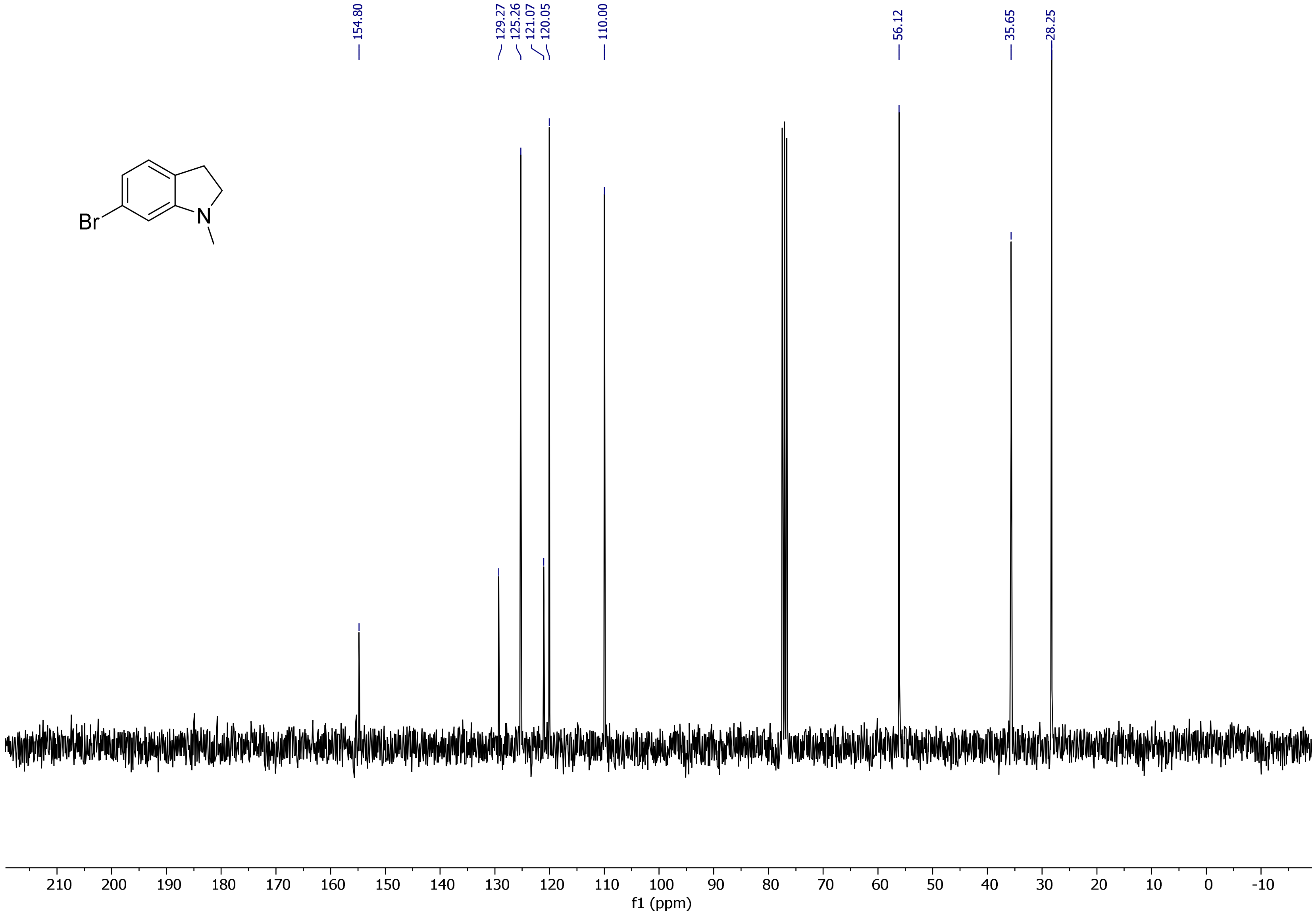

^{1}H NMR (300 MHz) in $CDCl_3$ for **2**

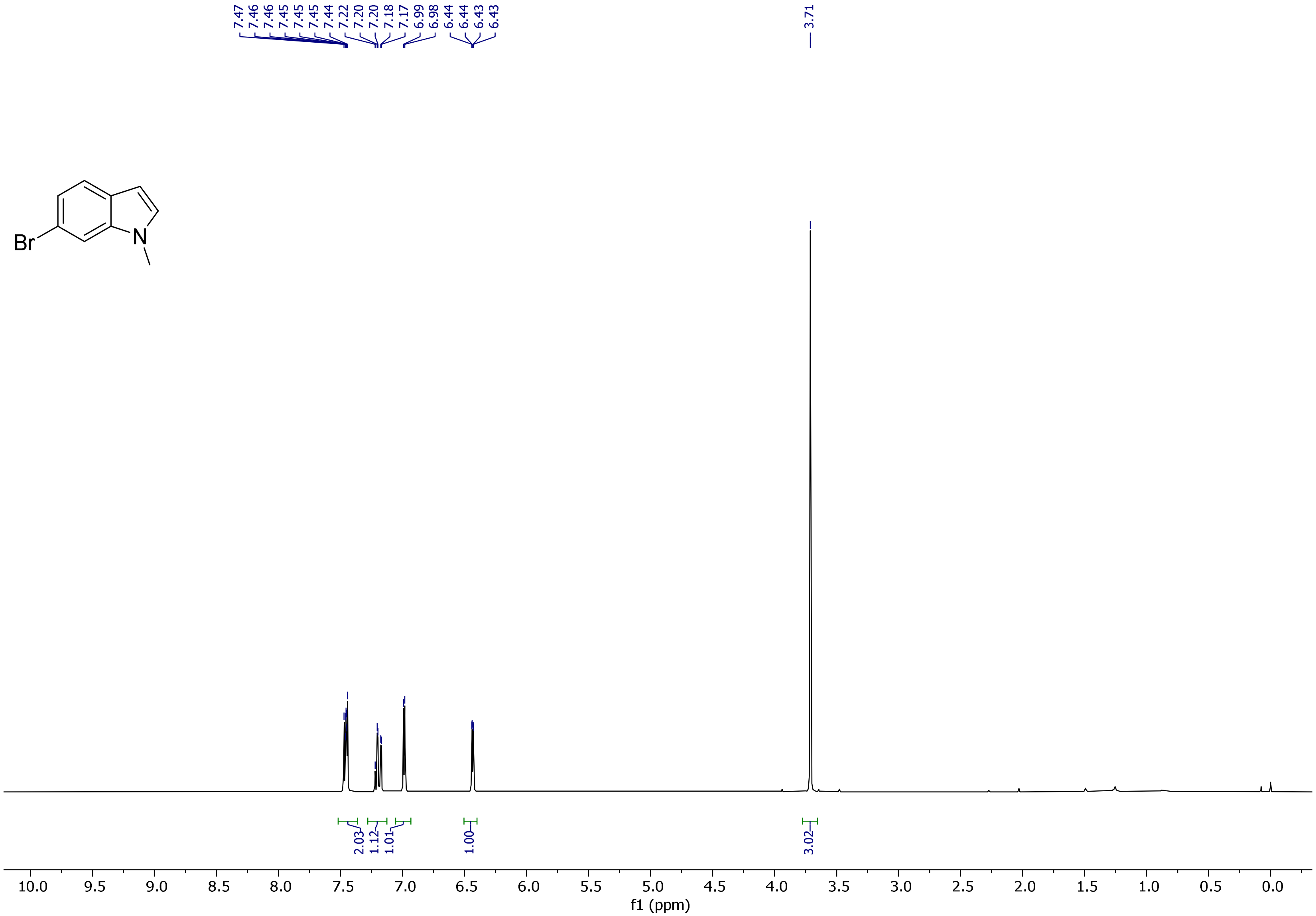

^{13}C NMR (75.5 MHz) in $CDCl_3$ for **2**

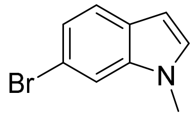

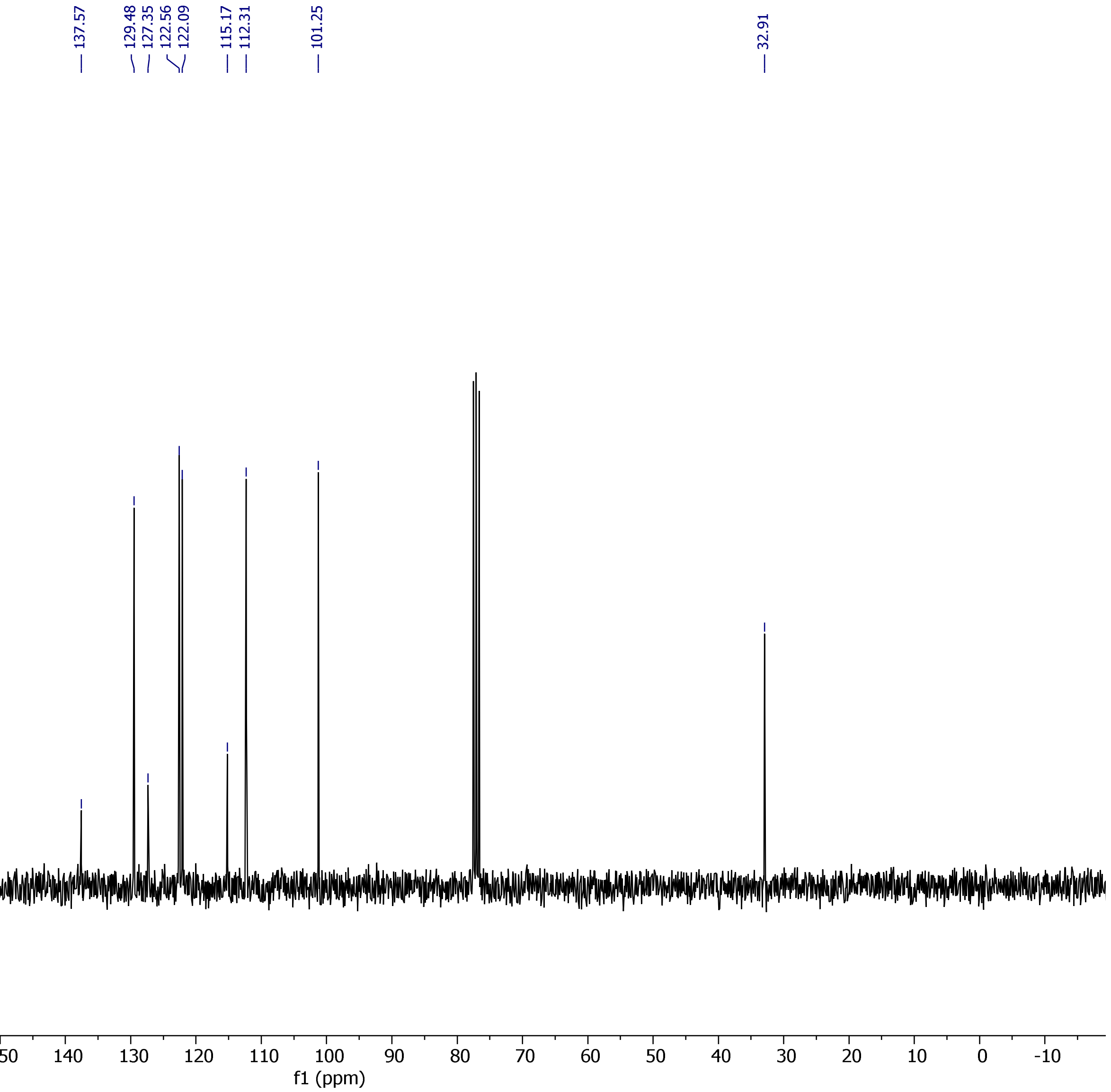

^{1}H NMR (300 MHz) in $CDCl_3$ for **4**

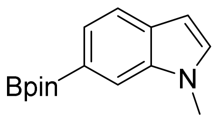

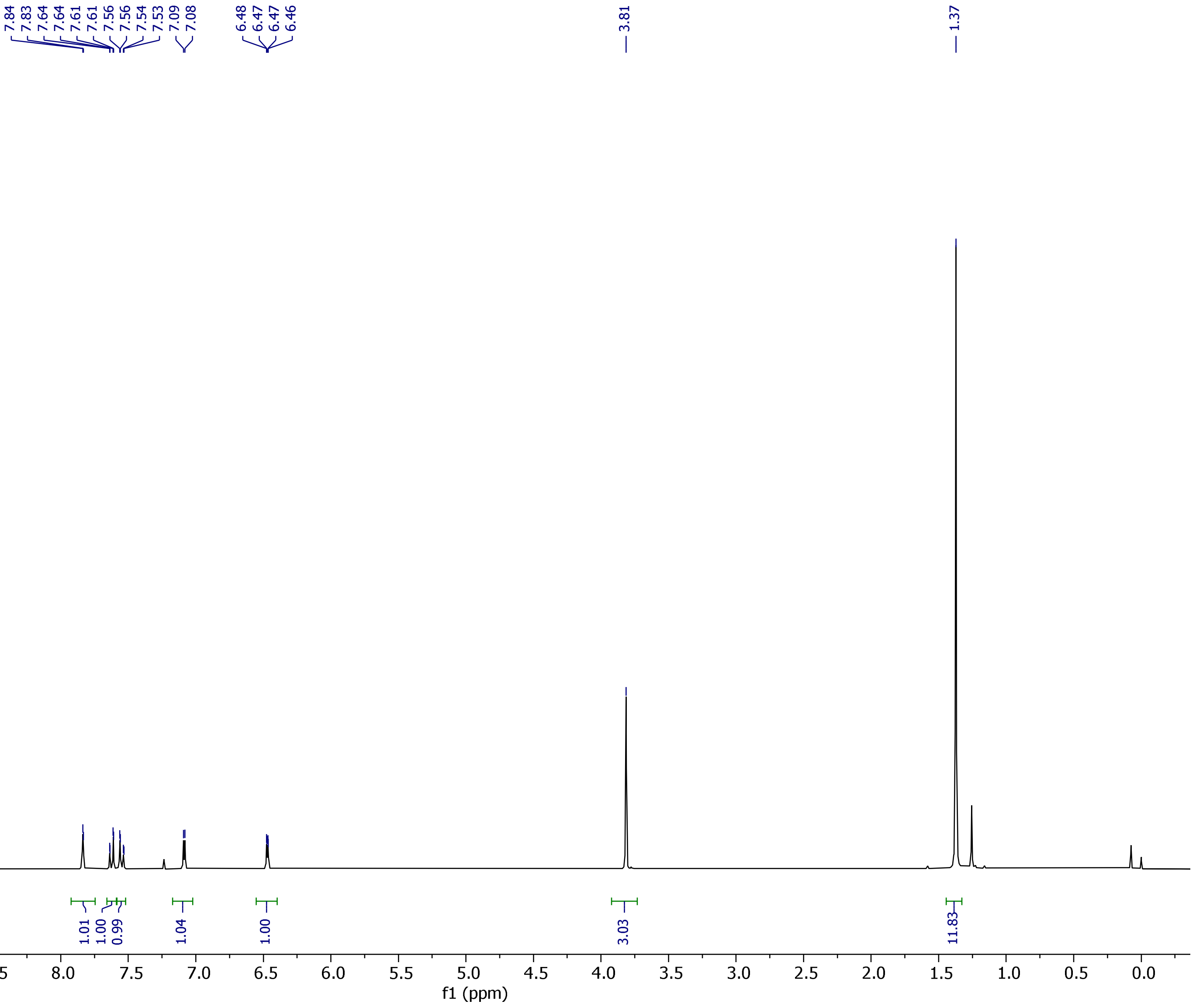

^{13}C NMR (75.5 MHz) in $CDCl_3$ for **4**

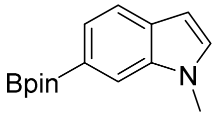

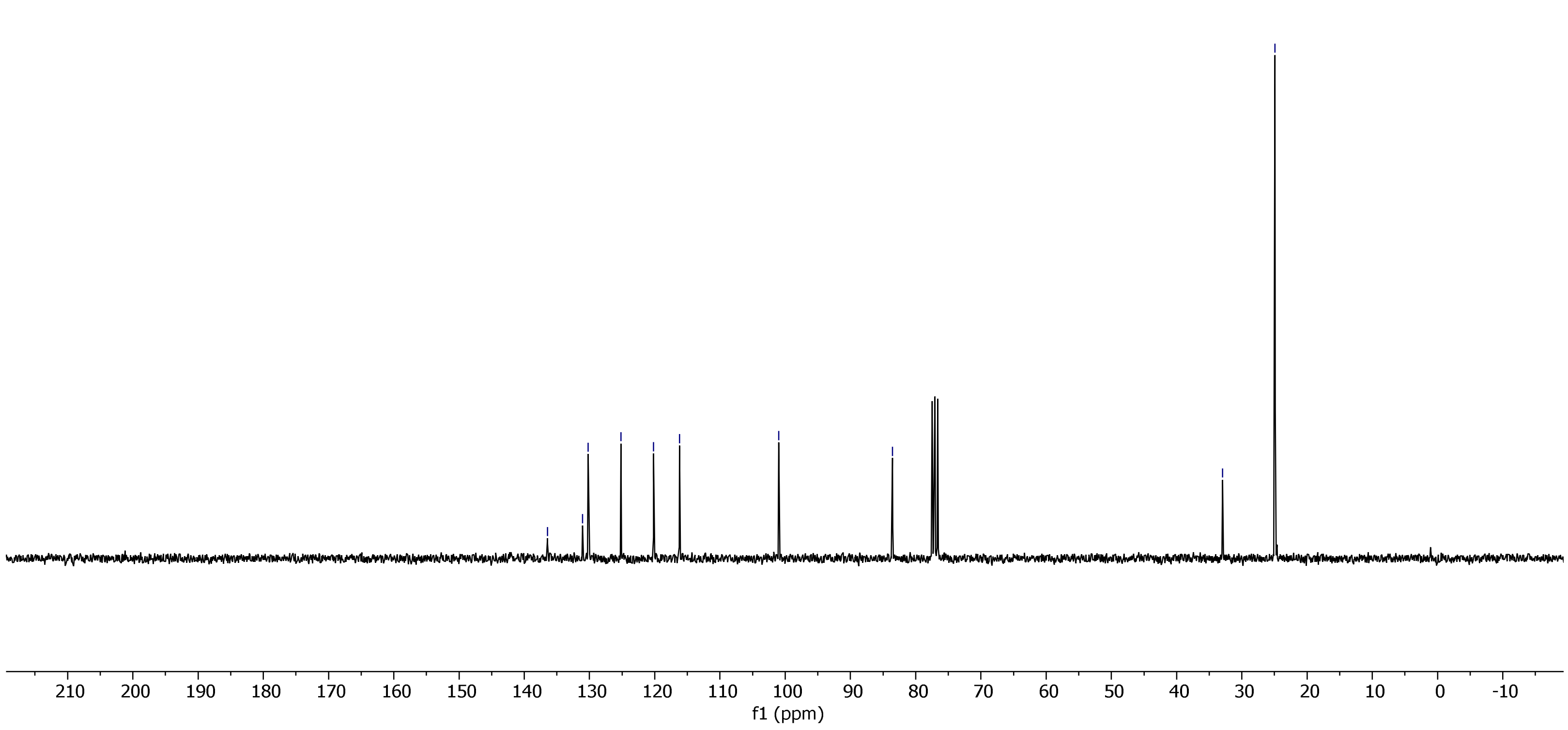

^{1}H NMR (300 MHz) in $CDCl_3$ for **3**

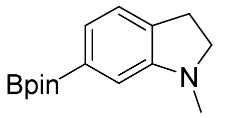

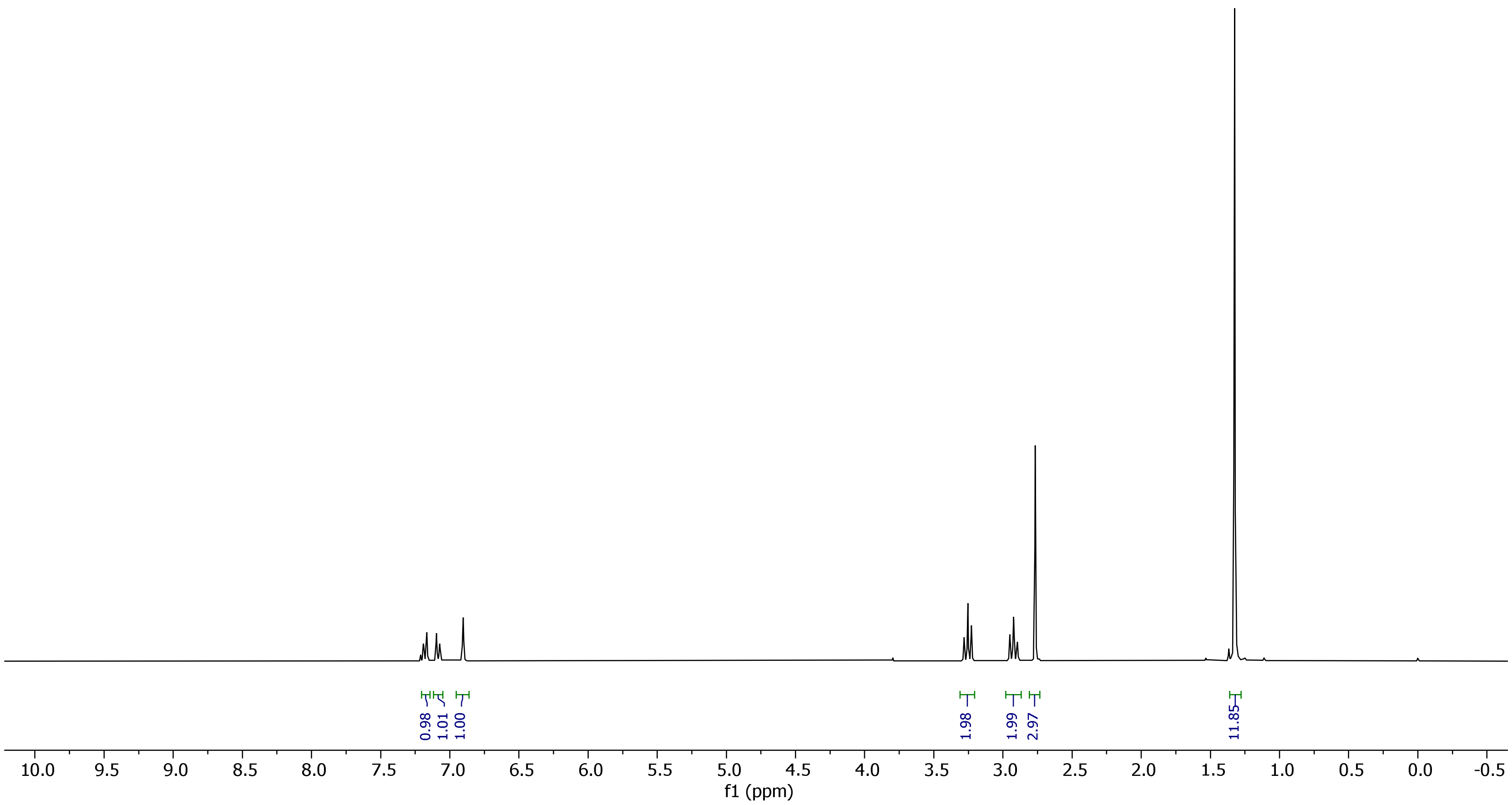

^{13}C NMR (75.5 MHz) in $CDCl_3$ for **3**

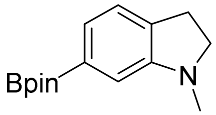

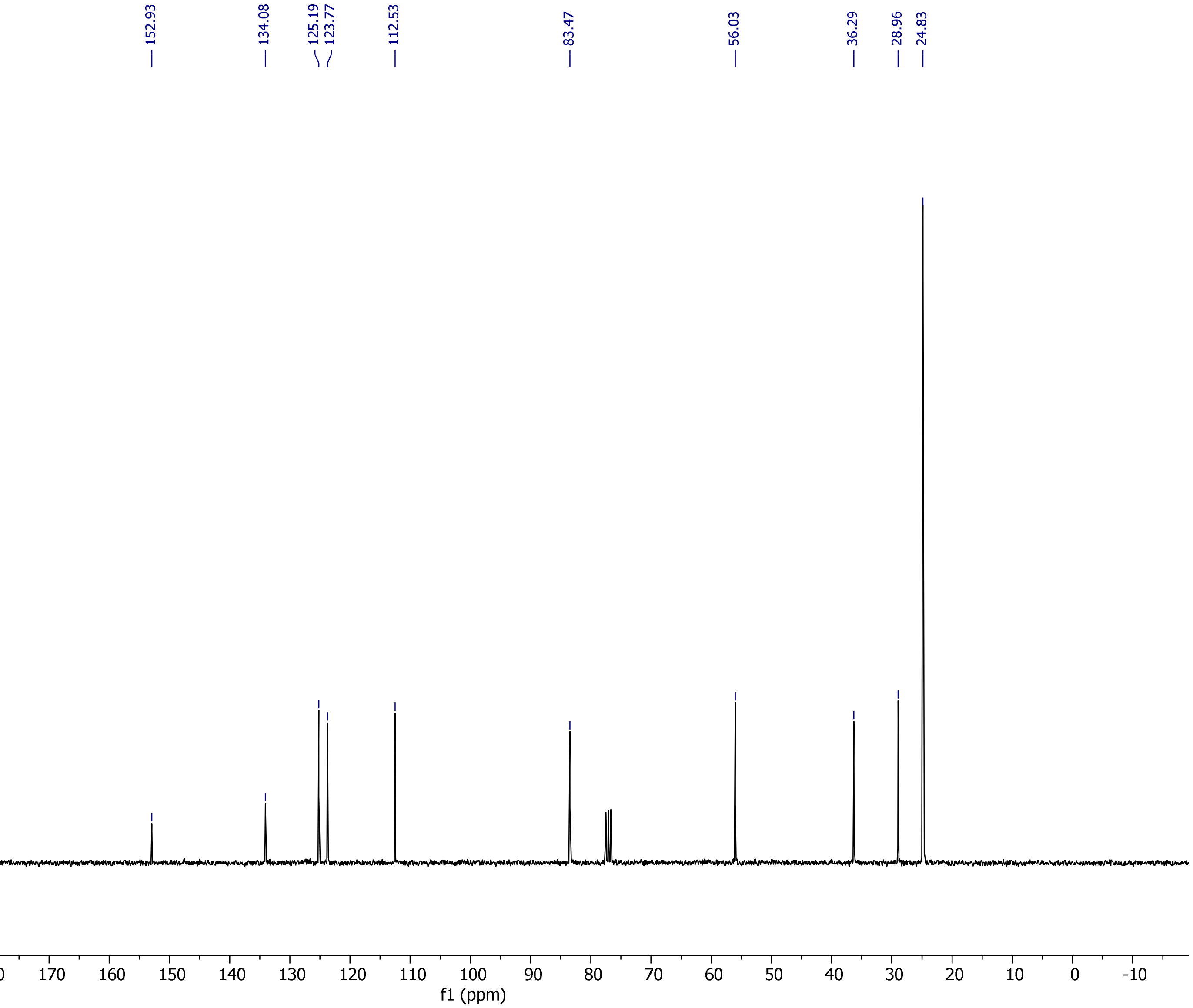

^{1}H NMR (300 MHz) in $CDCl_3$ for **14**

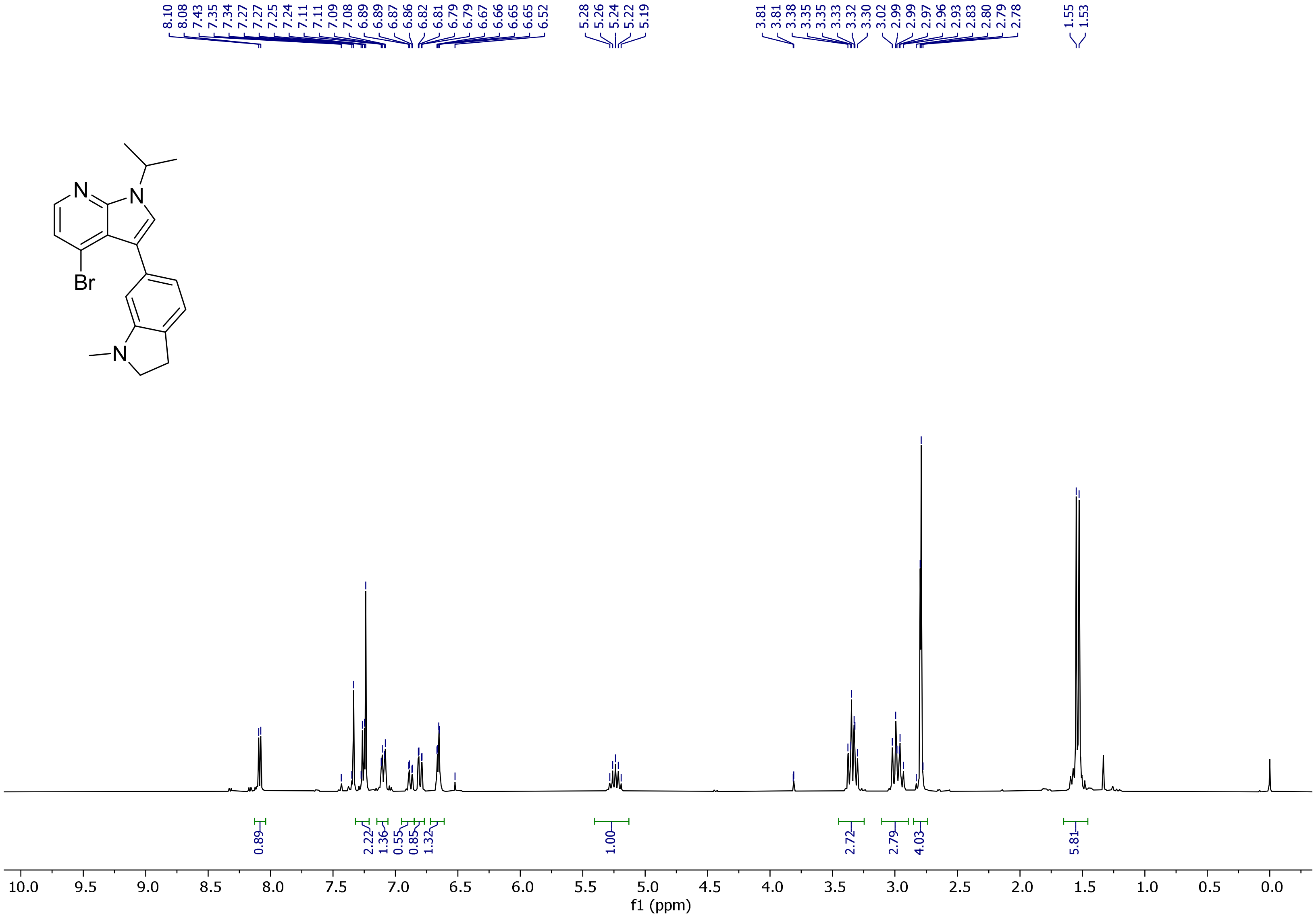

^{1}H NMR (300 MHz) in $CDCl_3$ for **15**

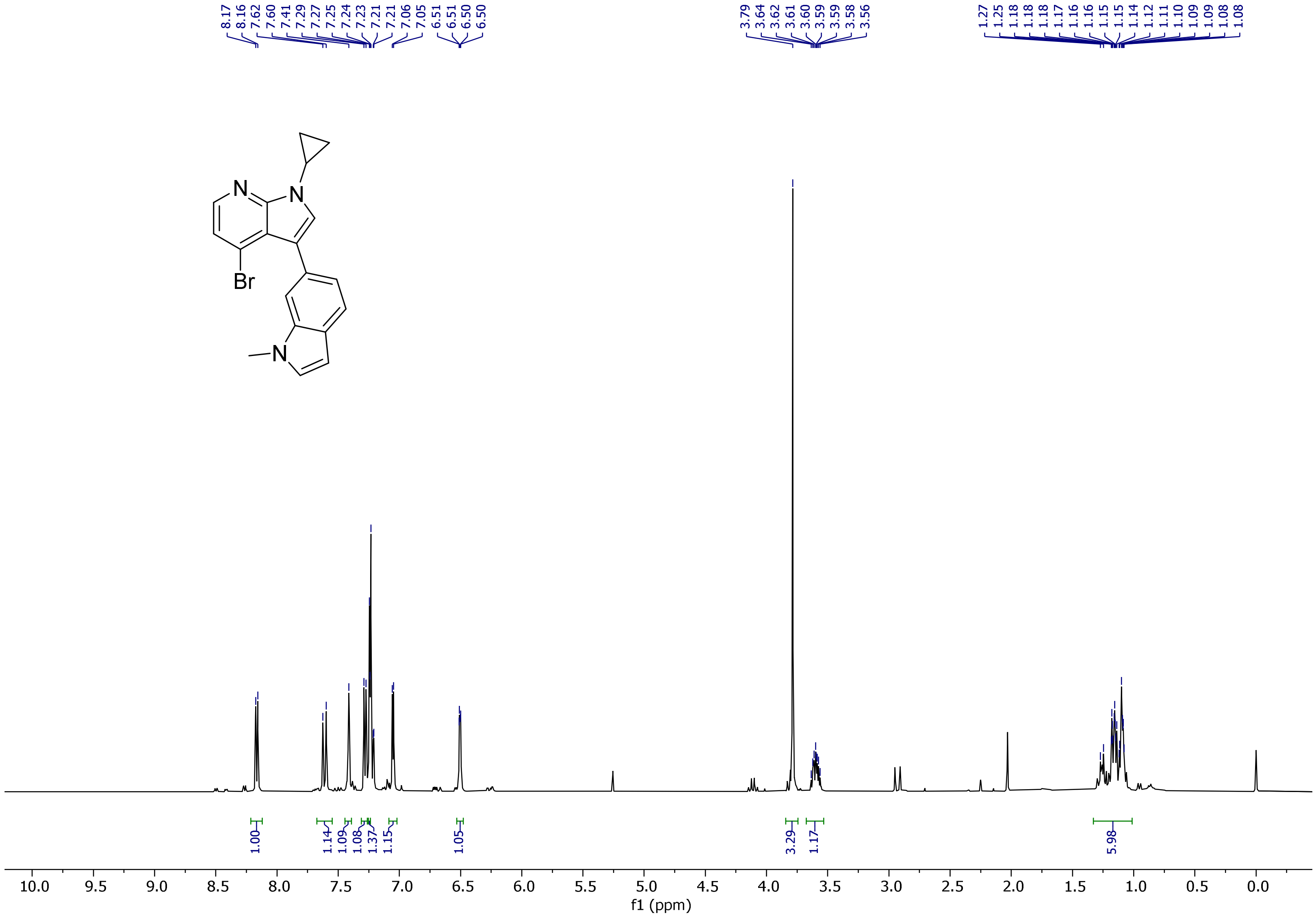

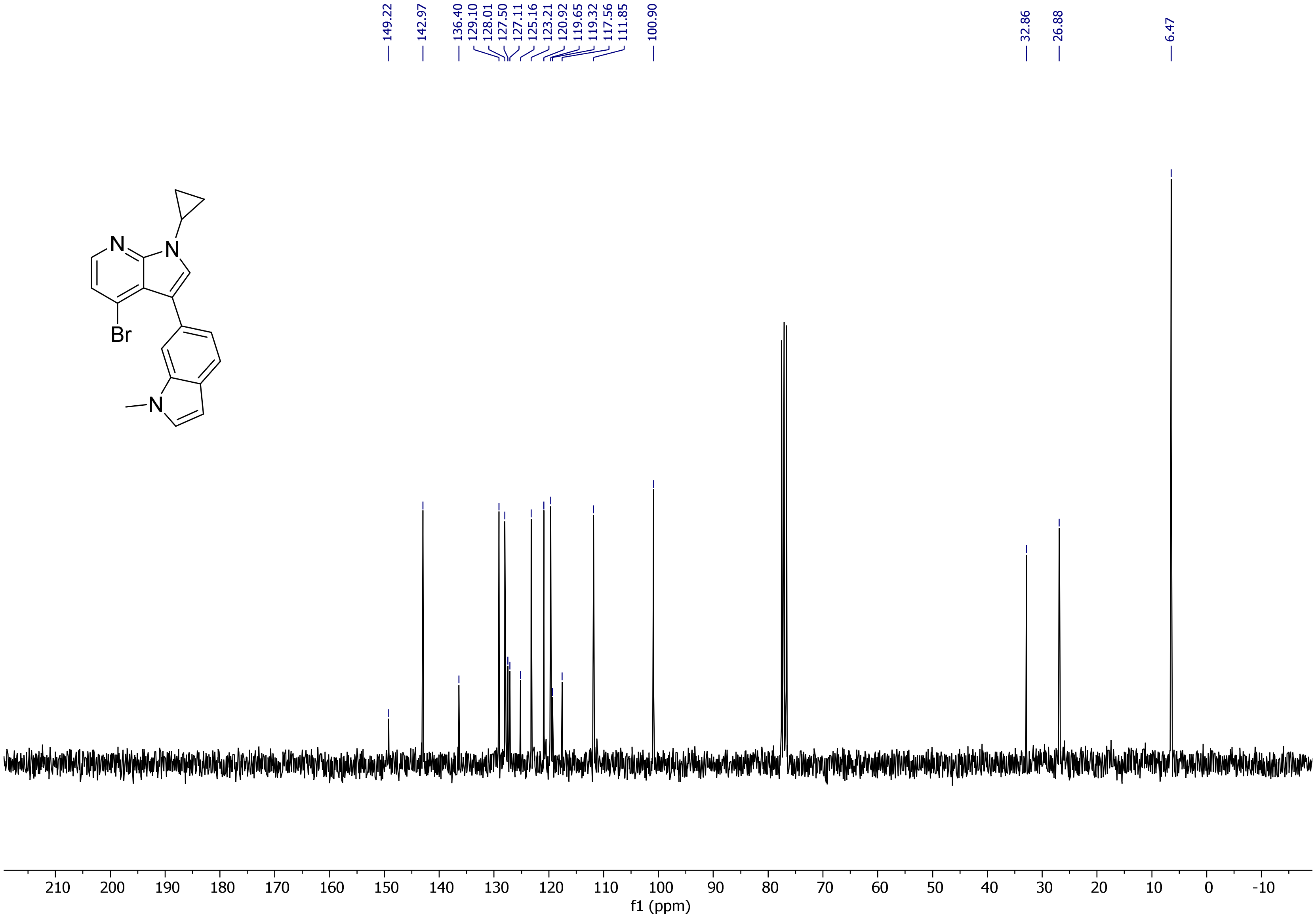
149.22
142.97
136.40
129.10
128.01
127.50
127.11
125.16
123.21
120.92
119.65
119.32
117.56
111.85
100.90
32.86
26.88
6.47
210
200
190
180
170
160
150
140
130
120
110
100
90
80
70
60
50
40
30
20
10
0
-10
f1 (ppm)

^{1}H NMR (300 MHz) in $CDCl_3$ for **16** (**GSK2795039**)

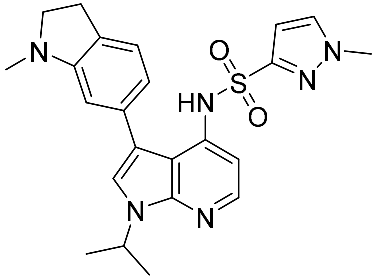

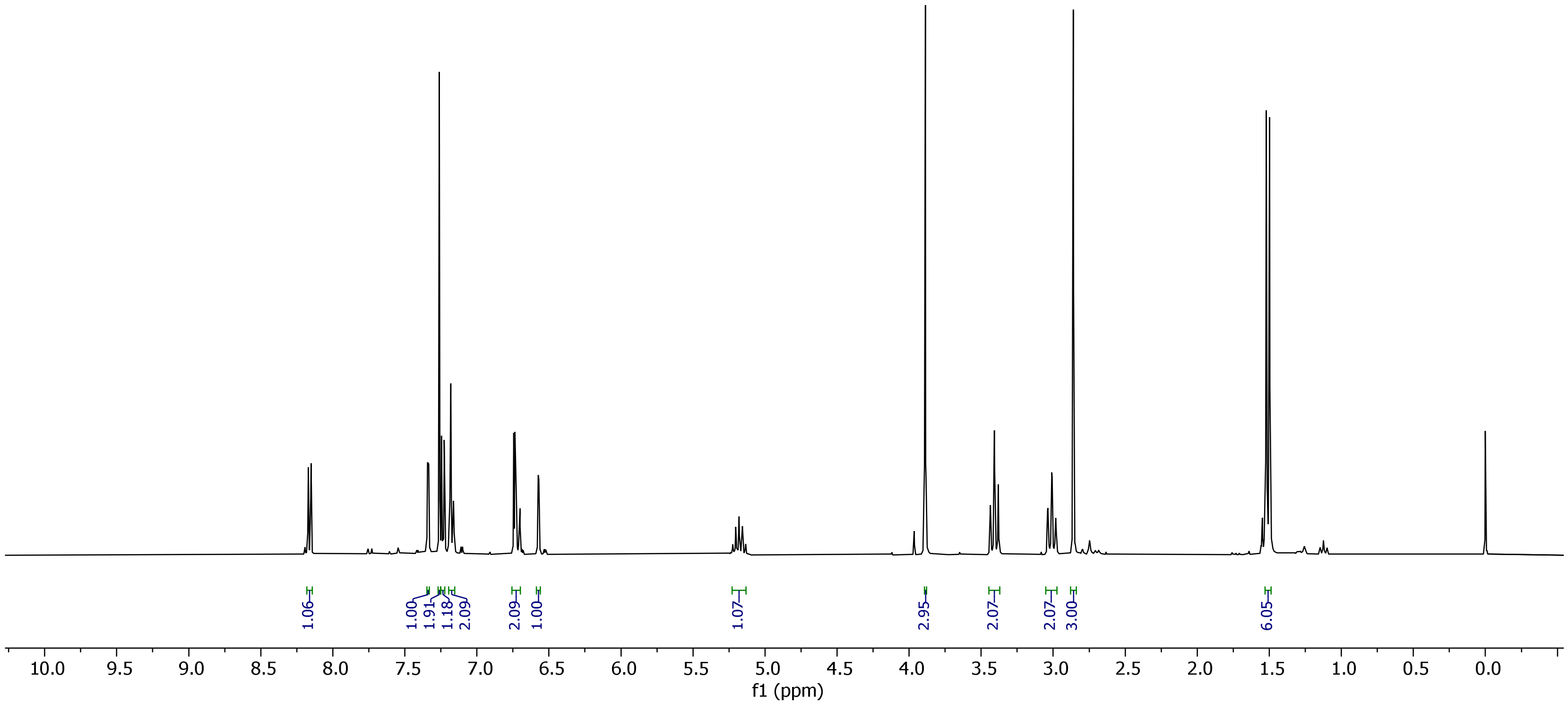

^{13}C NMR (75.5 MHz) in $CDCl_3$ for **16** (**GSK2795039**)

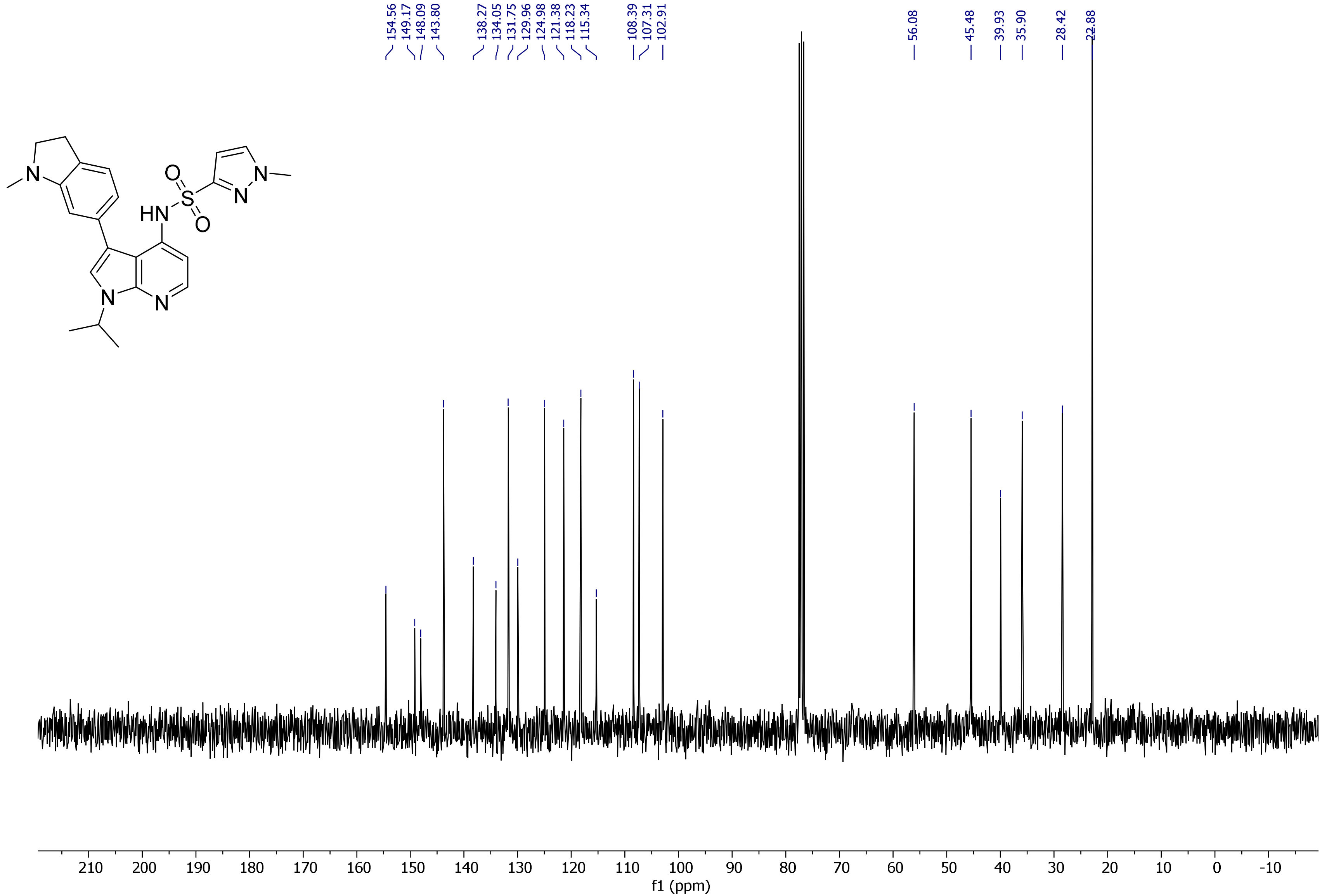

^{13}C-DEPT-135 NMR (75.5 MHz) in $CDCl_3$ for **16** (**GSK2795039**)

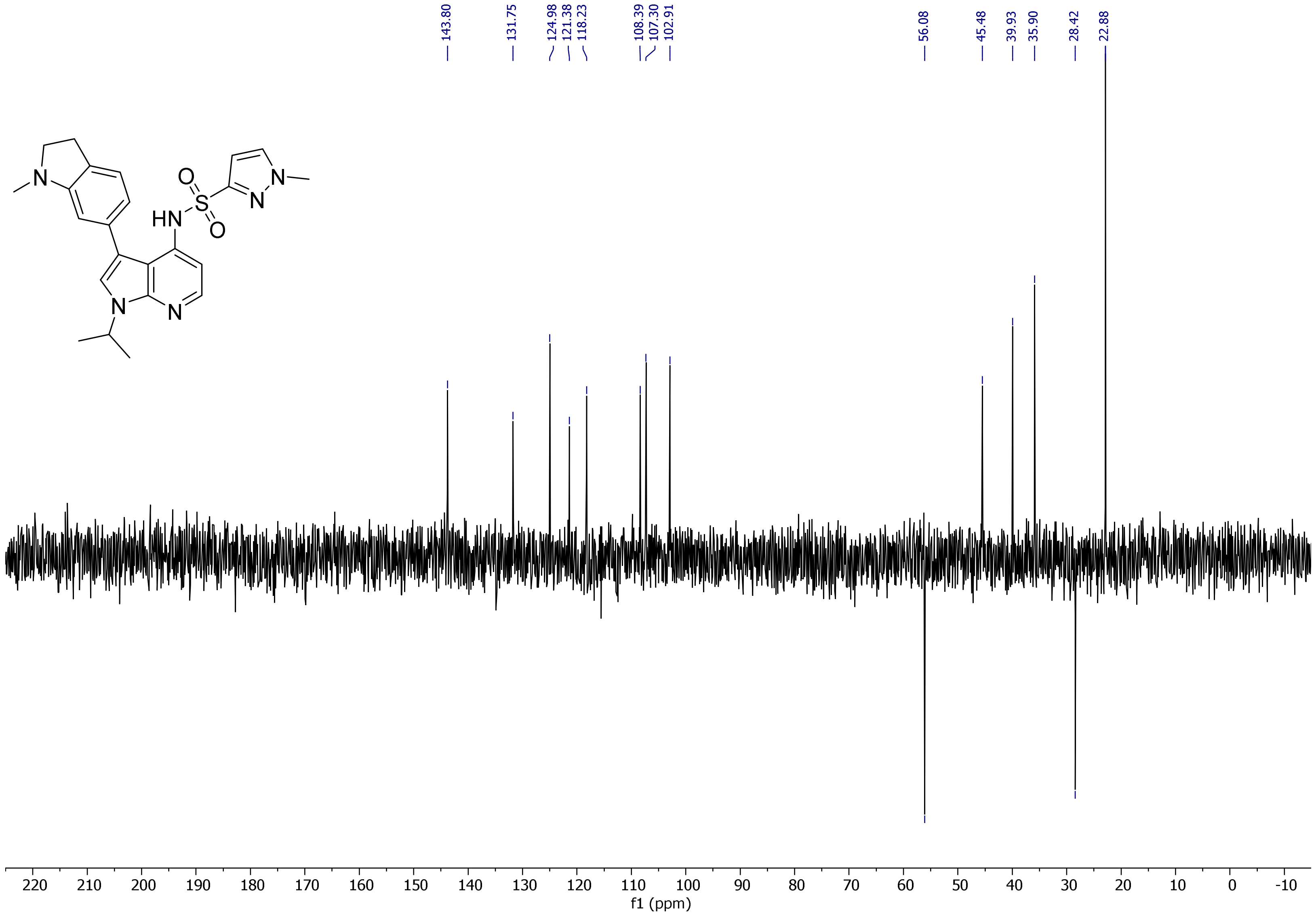

^{1}H NMR (300 MHz) in DMSO-d6 for **18** (**NCATS-SM7270**)

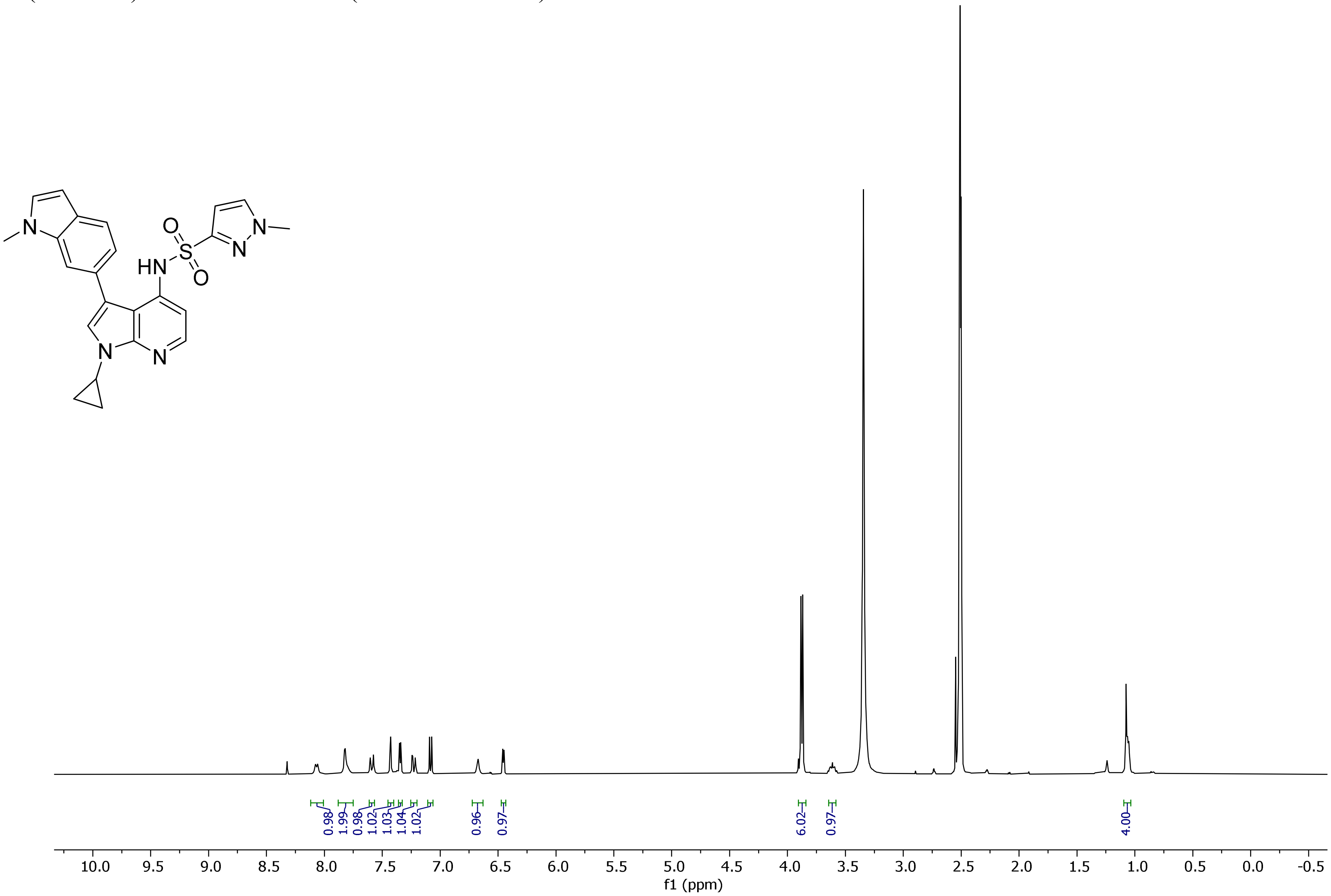

^{13}C NMR (75.5 MHz) in $CDCl_3$ for **18** (**NCATS-SM7270**)

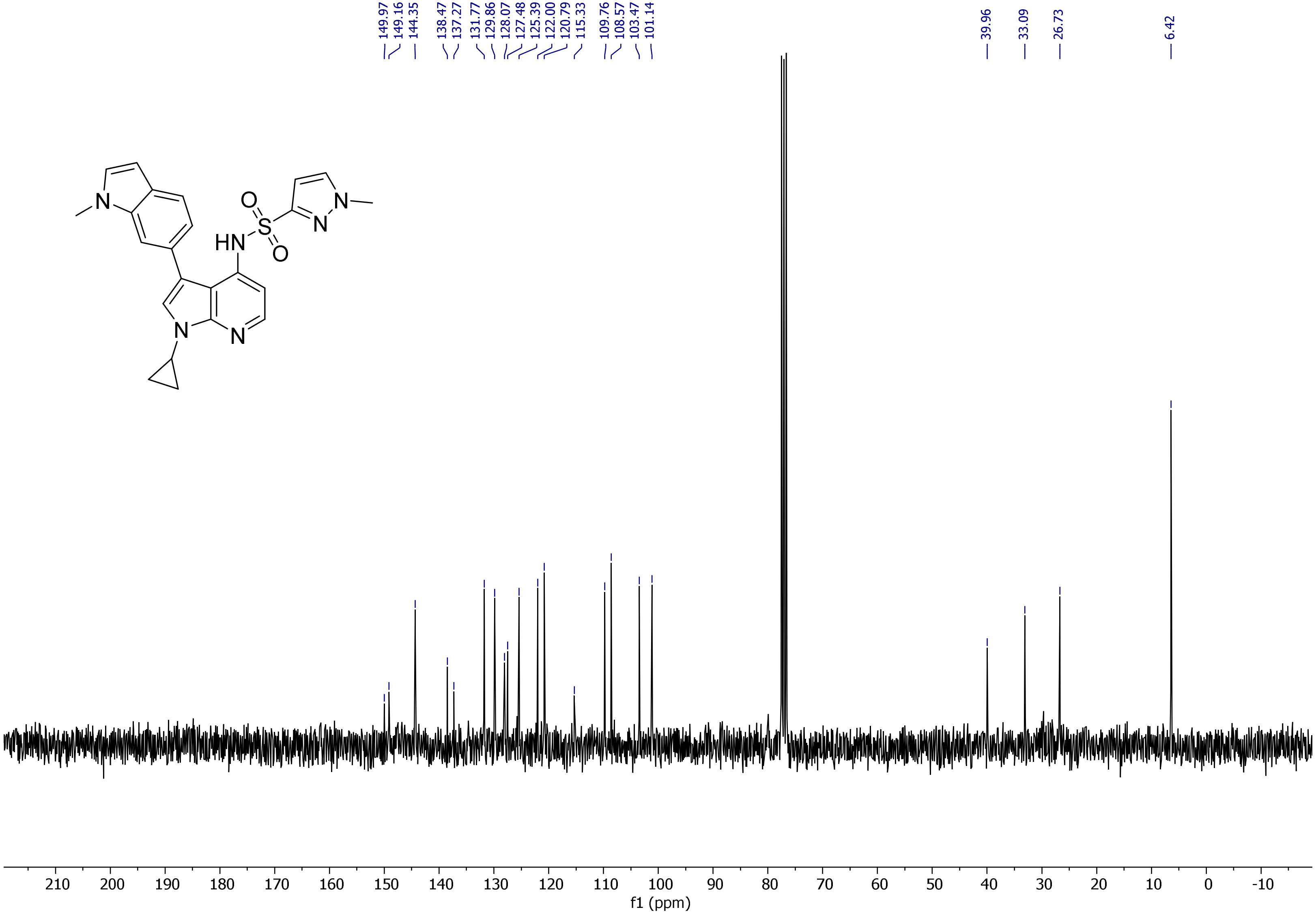

^{13}C-DEPT-135 NMR (75.5 MHz) in $CDCl_3$ for **18** (**NCATS-SM7270**)

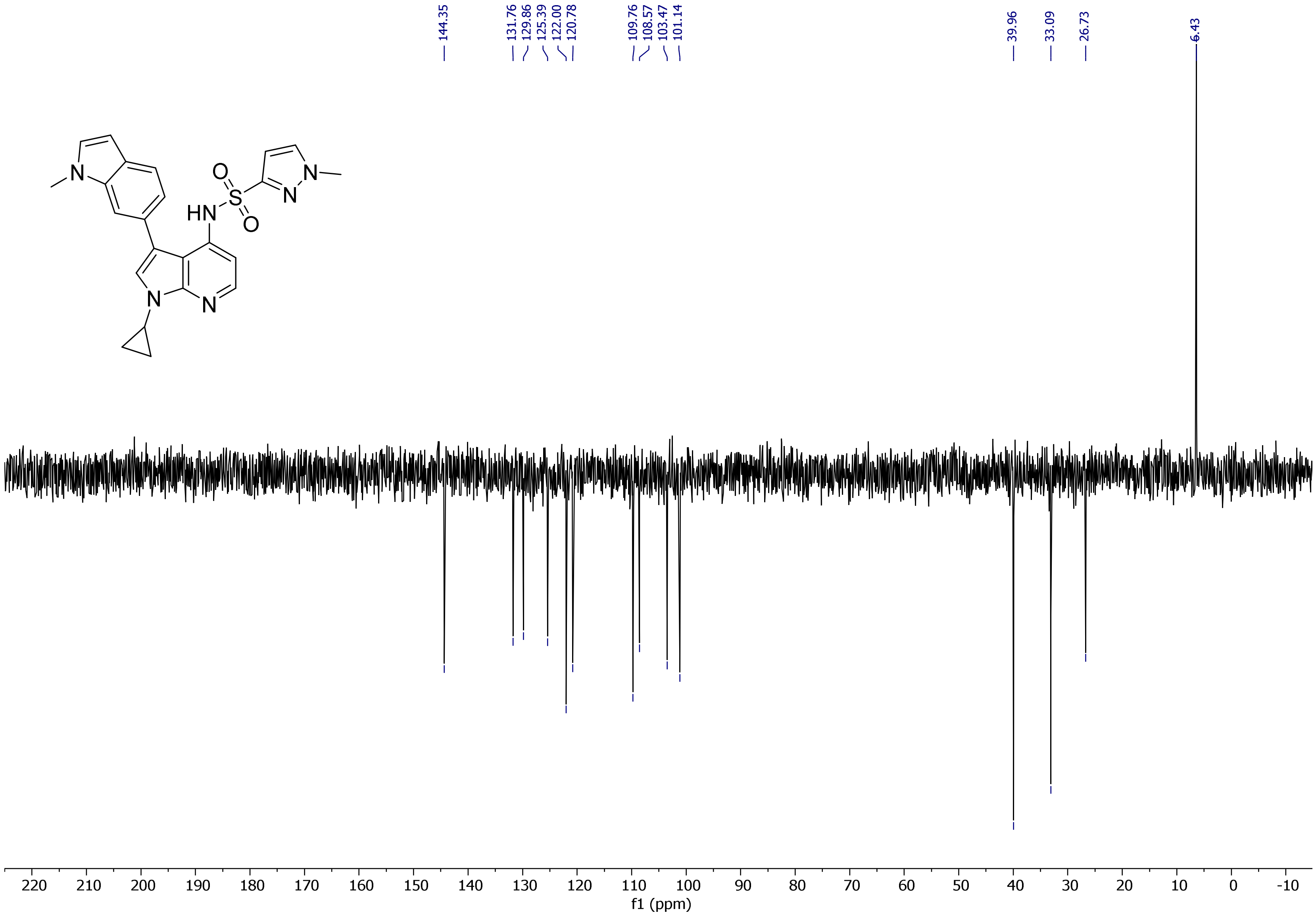

^{1}H,^{1}H-COSY (300 MHz) in $CDCl_3$ for **18** (**NCATS-SM7270**)

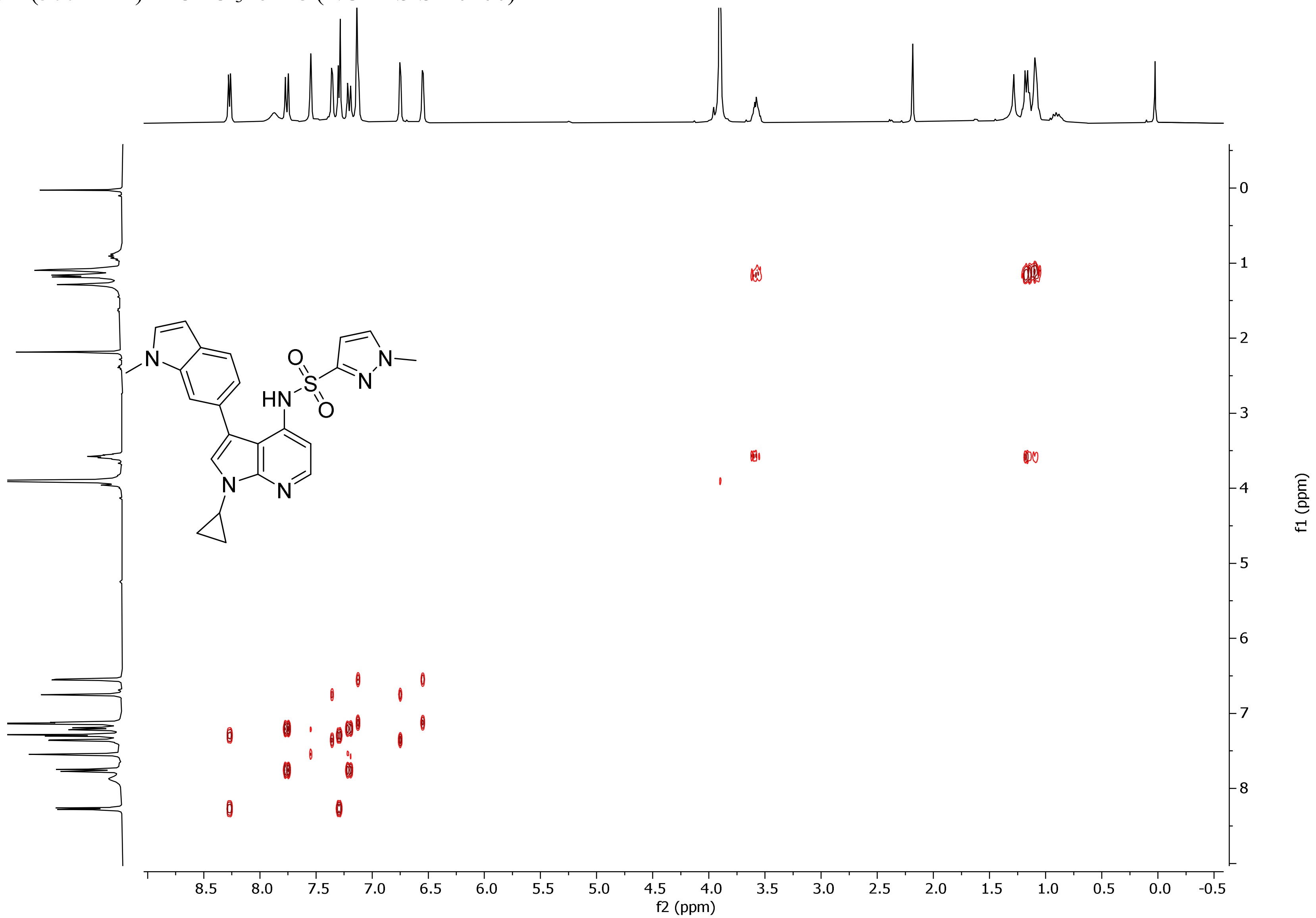

^{1}H,^{13}C-HSQC (300 and 75 MHz) in $CDCl_3$ for **18** (**NCATS-SM7270**)

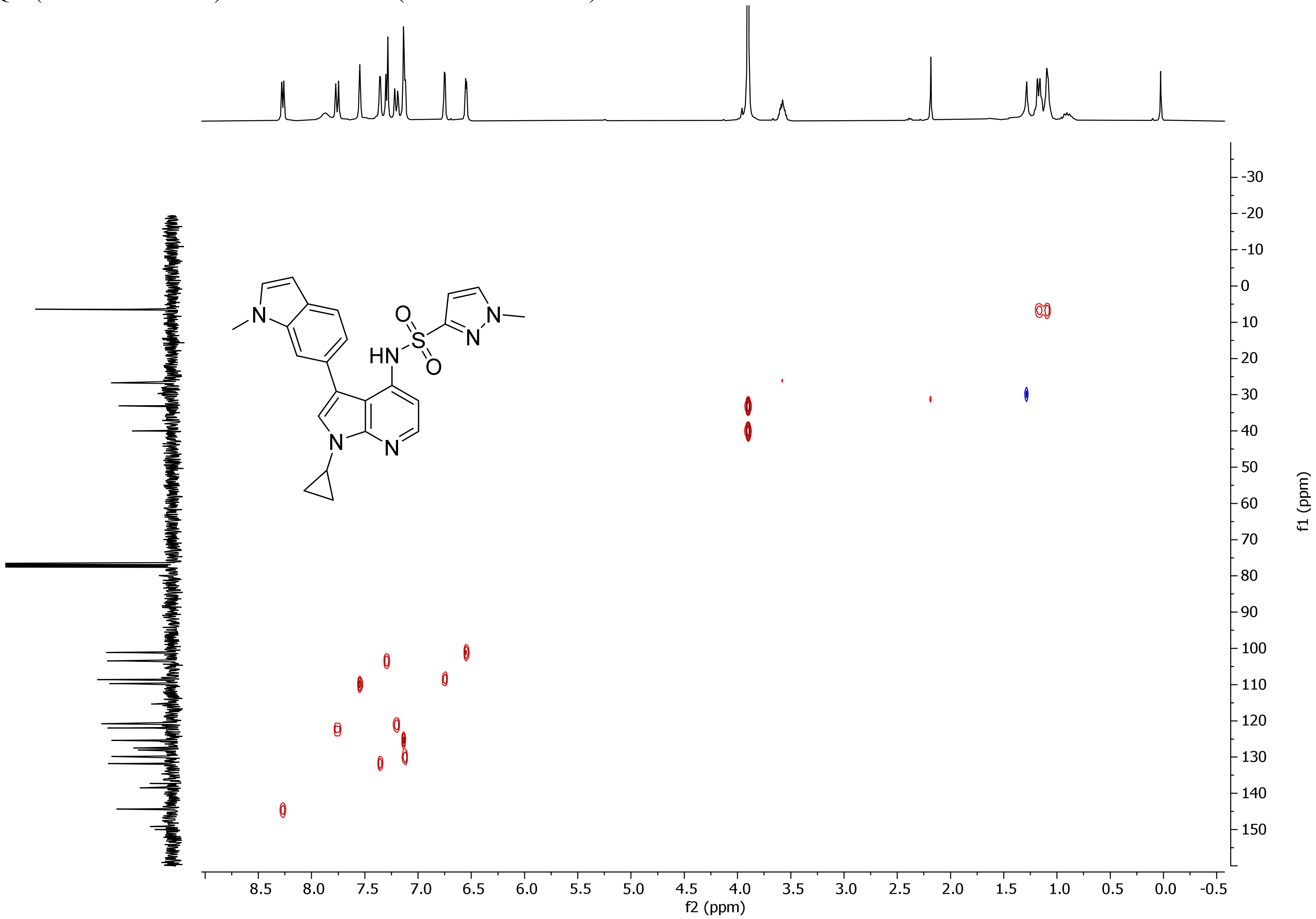

^{1}H NMR (300 MHz) in $CDCl_3$ for **17** (**IMBIOC-1**)

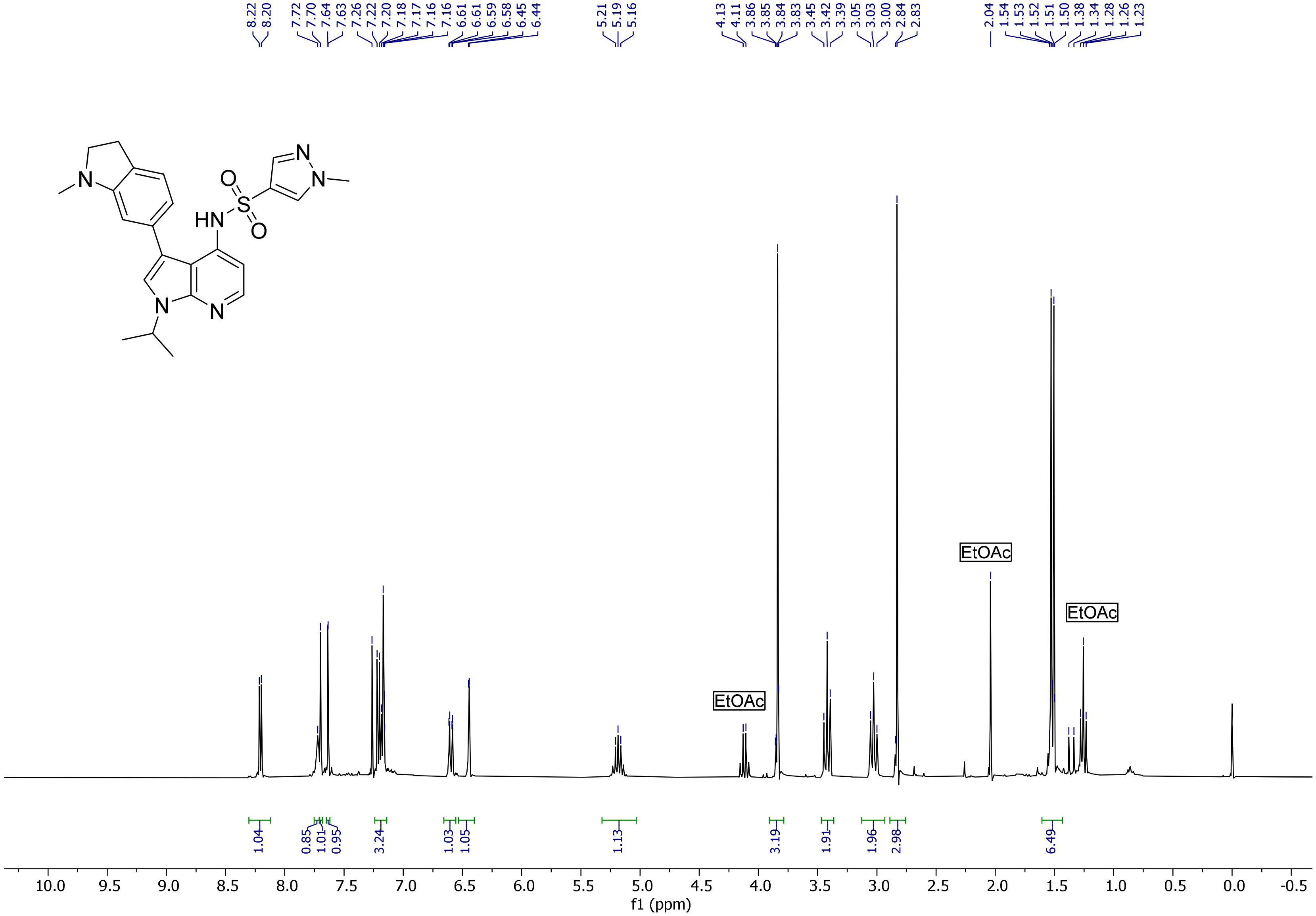

^{13}C NMR (75.5 MHz) in $CDCl_3$ for **17** (**IMBIOC-1**)

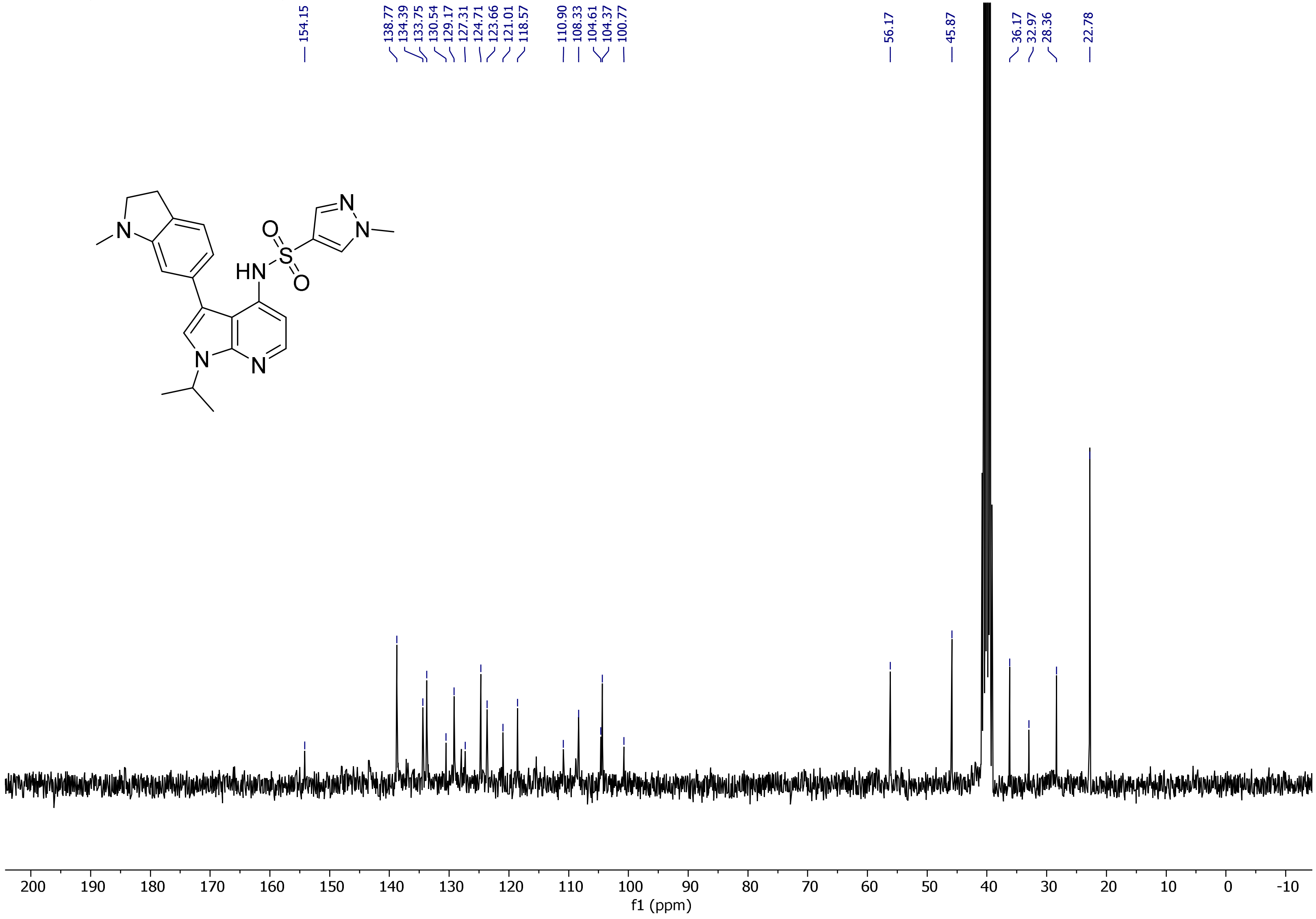

HPLC chromatogram and HRMS spectrum of **16** (GSK2795039).

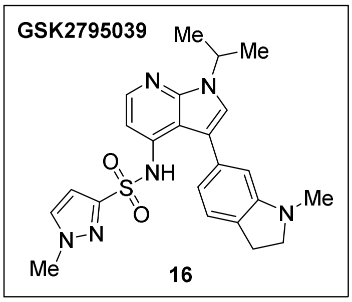

16

Display Report

Analysis Info

Analysis Name D:\Data\HOB-66 (GSK2795039)-_64_01_5199.d
Method la_2.2_small.m
Sample Name HOB-66 (GSK2795039)-
Comment

Acquisition Date 3/20/2025 2:04:37 PM

Operator BDAL@DE
Instrument compact 8255754.20088

Acquisition Parameter

Source Type	ESI	Ion Polarity	Positive	Set Nebulizer	0.4 Bar
Focus	Active	Set Capillary	4500 V	Set Dry Heater	180 C
Scan Begin	50 m/z	Set End Plate Offset	-500 V	Set Dry Gas	4.0 l/min
Scan End	3000 m/z	Set Charging Voltage	2000 V	Set Divert Valve	Source
		Set Corona	0 nA	Set APCI Heater	0 C

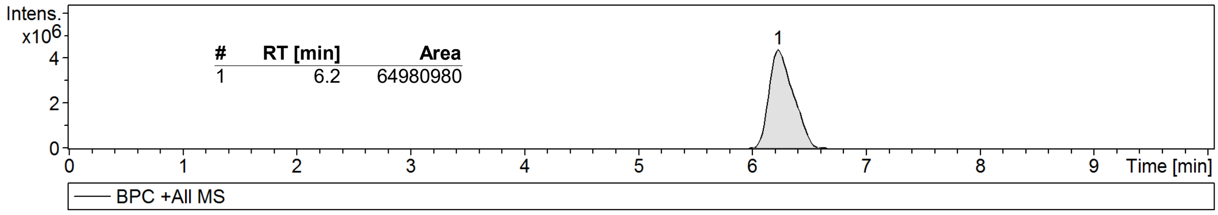

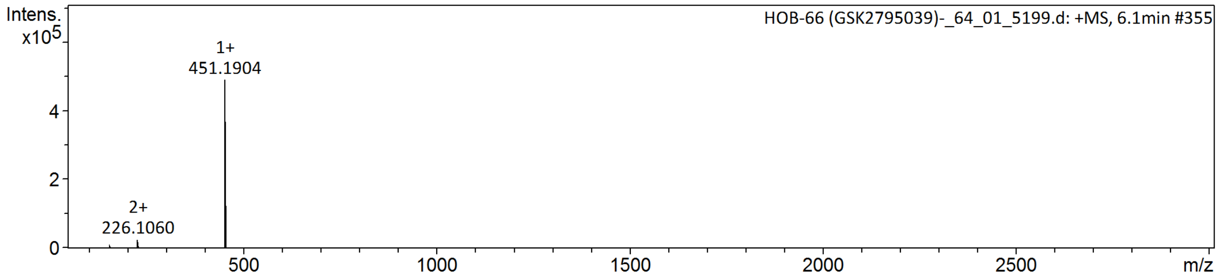

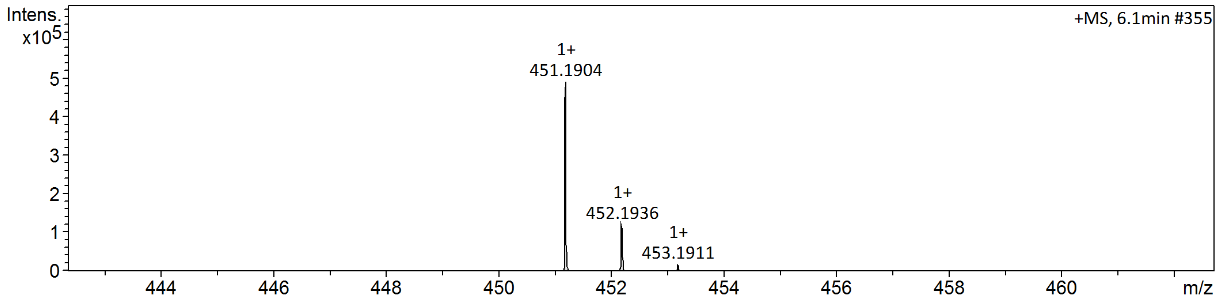

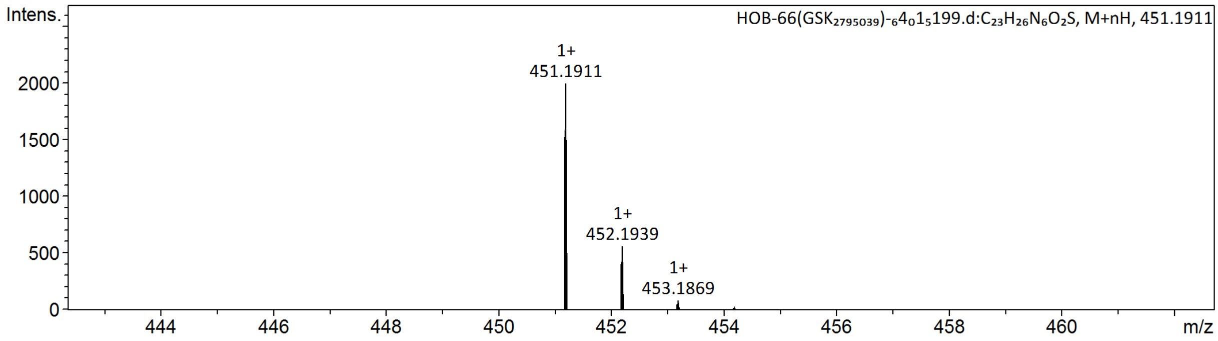

HPLC chromatogram and HRMS spectrum of **17** (IMBIOC-1).

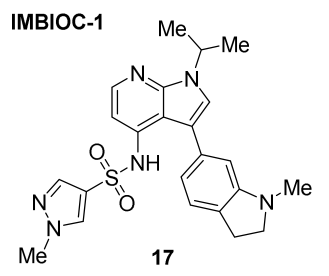

Display Report

Analysis Info

Analysis Name	D:\Data\HOB-64 (IMBIOC-1)_62_01_5197.d	Acquisition Date	3/20/2025 1:41:27 PM
Method	la_2.2_small.m	Operator	BDAL@DE
Sample Name	HOB-64 (IMBIOC-1)	Instrument	compact 8255754.20088
Comment			

Acquisition Parameter

Source Type	ESI	Ion Polarity	Positive	Set Nebulizer	0.4 Bar
Focus	Active	Set Capillary	4500 V	Set Dry Heater	180 C
Scan Begin	50 m/z	Set End Plate Offset	-500 V	Set Dry Gas	4.0 l/min
Scan End	3000 m/z	Set Charging Voltage	2000 V	Set Divert Valve	Source
		Set Corona	0 nA	Set APCI Heater	0 C

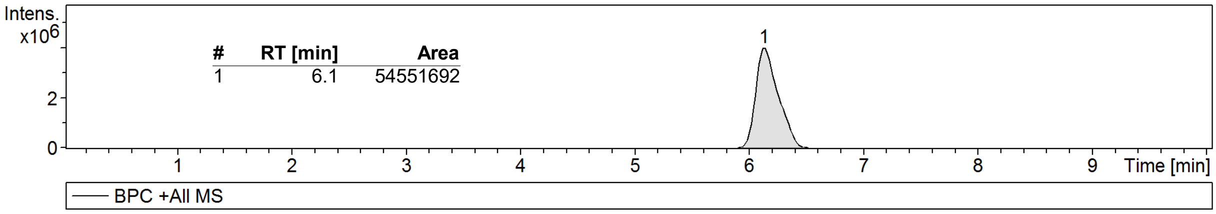

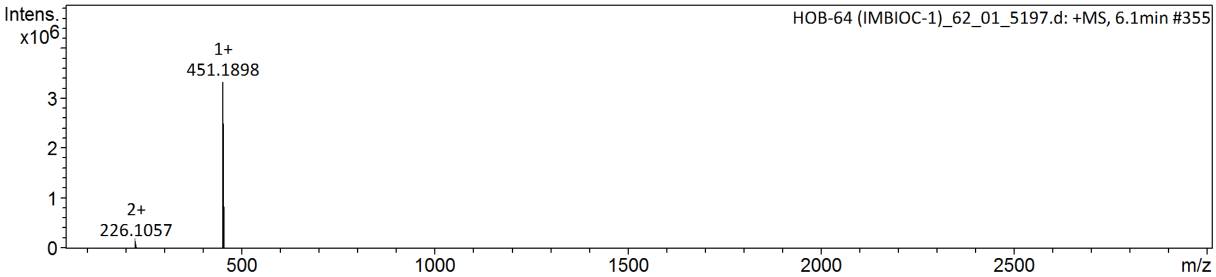

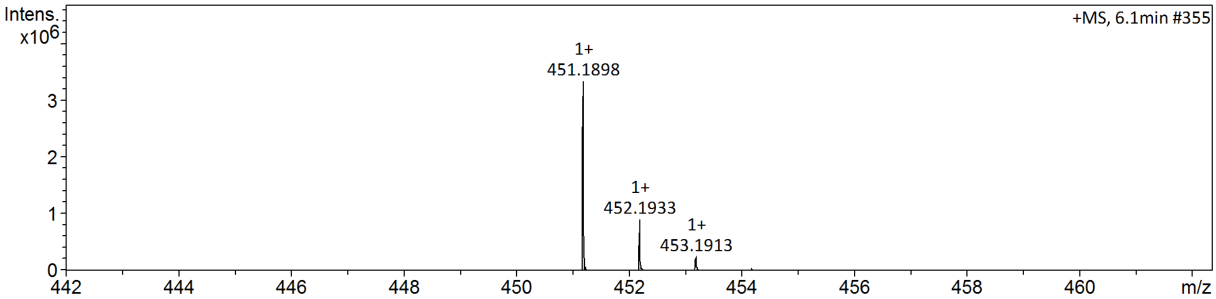

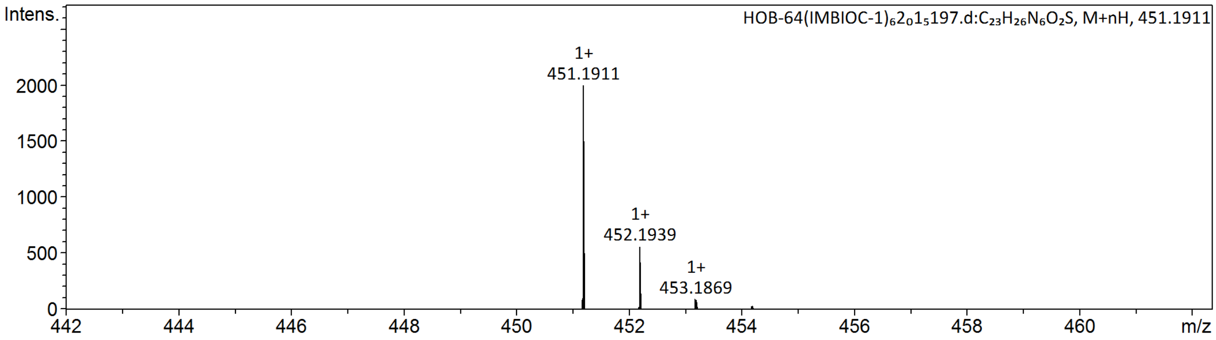

HPLC chromatogram and HRMS spectrum of **18** (NCATS-SM7270).

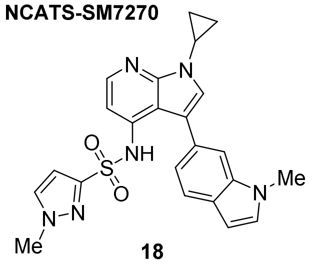

18

Display Report

Analysis Info

Analysis Name	D:\Data\Pavel\HOB-63 (NCATS-SM7270)_61_01_5196.d	Acquisition Date	3/20/2025 1:29:50 PM
Method	la_2.2_small.m	Operator	BDAL@DE
Sample Name	HOB-63 (NCATS-SM7270)	Instrument	compact 8255754.20088
Comment			

Acquisition Parameter

Source Type	ESI	Ion Polarity	Positive	Set Nebulizer	0.4 Bar
Focus	Active	Set Capillary	4500 V	Set Dry Heater	180 C
Scan Begin	50 m/z	Set End Plate Offset	-500 V	Set Dry Gas	4.0 l/min
Scan End	3000 m/z	Set Charging Voltage	2000 V	Set Divert Valve	Source
		Set Corona	0 nA	Set APCI Heater	0 C

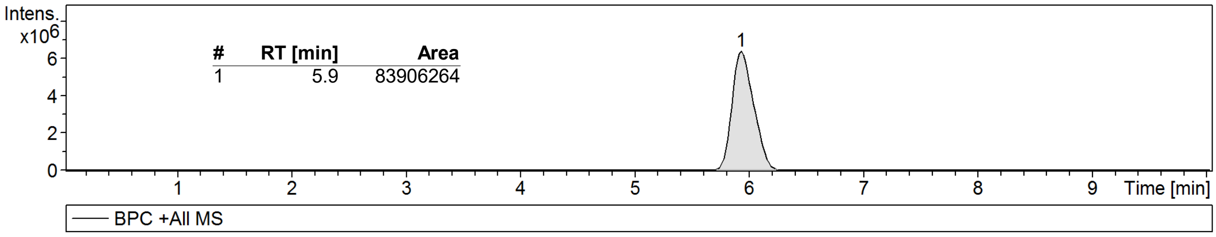

#	RT [min]	Area
1	5.9	83906264

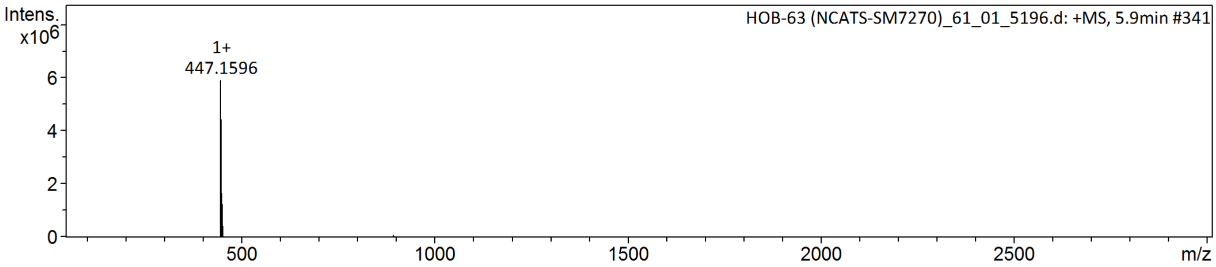

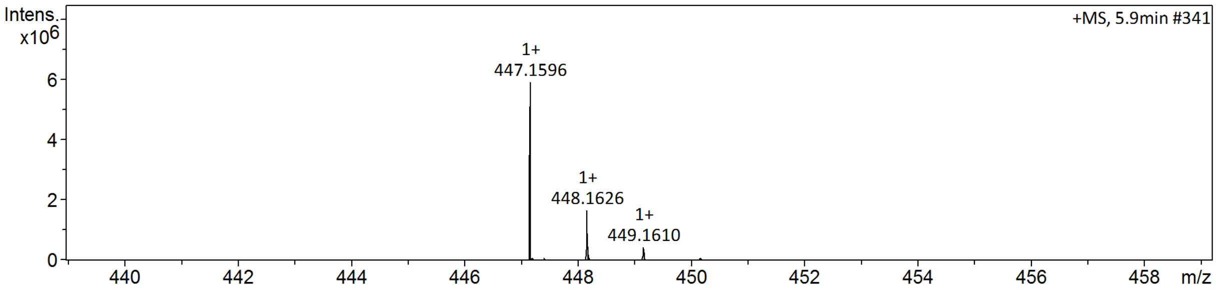

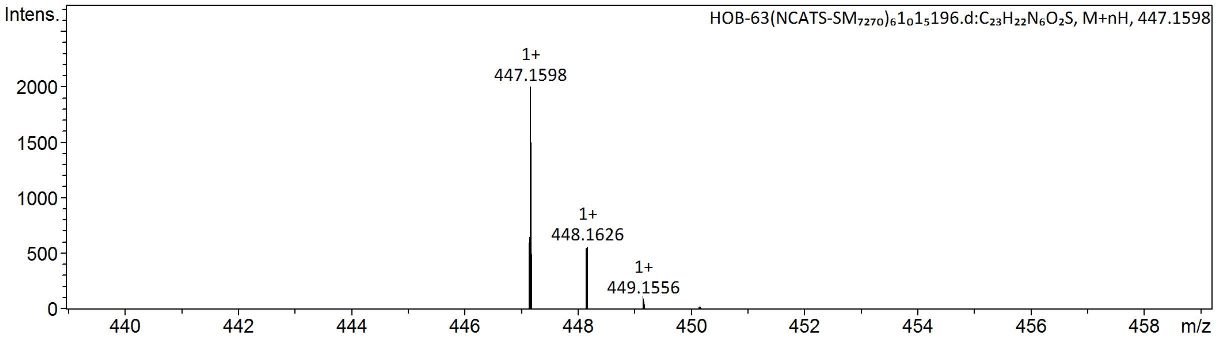

HOB-63 (NCATS-SM7270)_61_01_5196.d
